# Cancer and cancer survival modulates brain and behavior in a time-of-day-dependent manner in mice

**DOI:** 10.1038/s41598-019-42880-w

**Published:** 2019-04-24

**Authors:** Jessica C. Santos, Savannah R. Bever, Kyle A. Sullivan, Leah M. Pyter

**Affiliations:** 1 Institute for Behavioral Medicine Research, Ohio State University Wexner Medical Center, Columbus, OH USA; 2 Postgraduate Program in Basic and Applied Immunology, Ribeirão Preto School of Medicine, University of São Paulo, Ribeirão Preto, SP Brazil; 3 Department of Psychiatry and Behavioral Health, Ohio State University, Columbus, OH USA; 4 Department of Neuroscience, Ohio State University, Columbus, OH USA

**Keywords:** Microglia, Cognitive neuroscience, Neuroimmunology, Breast cancer

## Abstract

Improvements in breast cancer therapy/diagnosis have substantially increased the cancer survivor population, although many survivors report persistent mental health issues including fatigue, mood and anxiety disorders, and cognitive impairments. These behavioral symptoms impair quality-of-life and are often associated with increased inflammation. Nocturnal rodent models of cancer are critical to the identification of the neurobiological mechanisms underlying these behavioral changes. Although both behavior and immunity display distinct diurnal patterns, most rodent research in this field is performed during the rodents’ inactive (light) period, which could potentially undermine the conclusions and clinical relevance. Therefore, here we tested the extent to which mammary tumors or tumor resection (“survivors”) in mice affects behavior and neuroinflammation in a nyctohemeral (day versus night)-dependent manner. Indeed, only the dark (active) phase unmasked fatigue-like behavior and altered novel object investigation for both tumor-bearing and -resected mice relative to surgical controls. Several inflammatory markers were expressed in a time-of-day-dependent manner (lower in the dark phase) in the blood and brains of surgical control mice, whereas this temporal pattern was absent (IL-1β, CXCL1, *Myd88*, *Cd4*) or reversed (*C3*) in the respective tissues of tumor-bearing and -resected mice. Taken together, these data indicate that the time of day of assessment significantly modulates various persistent and transient tumor-induced behavioral and immune changes.

## Introduction

The time of day that behavioral assessments occur affects performance outcomes in rodent and human research, with enhanced locomotion and cognitive performance occurring during their respective active phases^1,2^. Thus, diurnal animals (e.g., humans) perform better when tested during the day and nocturnal animals (e.g., mice) perform better when tested during the night or dark phase of their daily laboratory light cycle. In some rodent cancer models, tumors induce depressive- and anxiety-like behavior, as well as cognitive impairments^3–9^, whereas in other models, reports of behavioral changes are lacking^3,4,10–13^. In parallel, clinical reports indicate that similar behavioral issues in cancer patients arise prior to chemotherapy or other cancer treatments, suggesting that tumor biology may contribute to the behavioral changes (reviewed in^14^). Indeed, significant evidence indicates that peripheral tumors affect brain function through peripheral-to-brain inflammatory pathways^3–8^. Of note, these behavioral changes can persist for many years in cancer survivors^15–17^.

Nevertheless, in nocturnal rodent cancer models typical behavioral testing occurs at a temporally and visually convenient time for researchers: during the rodents’ inactive, resting period (light phase)^18–20^. Thus, these behavioral effects of tumors may be difficult to interpret, underestimated, and/or poorly translational. Indeed, discrepancies in the timing of assessments may, in part, explain some inconsistencies in the findings. Additionally, putative underlying mechanisms, including inflammation and general immune function, exhibit circadian rhythmicity^21^, with significant increases in circulating immune cells and inflammatory cytokines as well as microglial activity in the brain during the resting phase (dark phase for humans, light phase for nocturnal rodents)^22^. In clinical cancer studies, behavior is typically assessed during peak performance, whereas immune assessments occur during circadian immune troughs; the opposite timing typically occurs for studies using rodent models of cancer. Thus, the time of day of assessments may significantly impact the interpretation of data and conclusions about the relationship between inflammation and behavioral changes associated with tumors. Here, we sought to identify potential behavioral and neurobiological effects of tumors that are dependent on the time of day. We hypothesized that tumors induce greater neuroinflammation during the light (inactive) phase, but greater behavioral changes during the dark (active) phase, in nocturnal mice.

## Results

### Effects of tumors and tumor resection on gross tissue masses were independent of time of day

**Figure 1:**
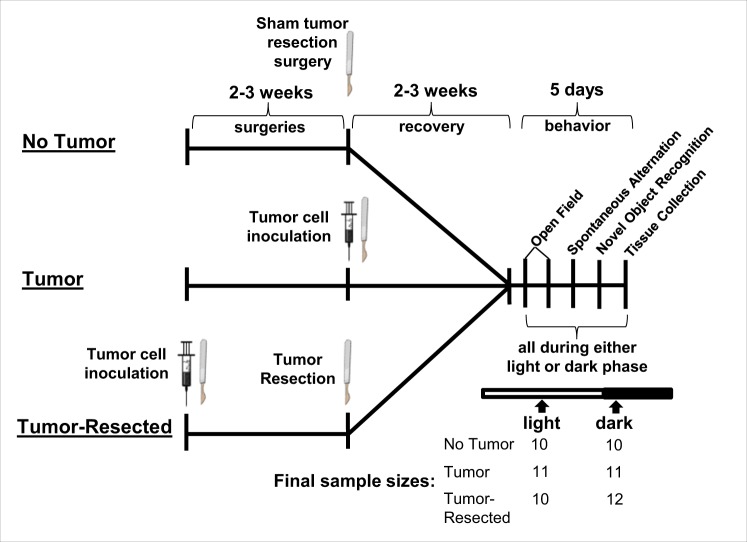
Experimental timeline and sample sizes for testing how time of day modulates behavioral and neuroinflammatory changes in tumor-free (surgical controls), tumor-bearing, and tumor-resected mice. Tumor-resected mice were surgically inoculated with mammary tumor cells. Between 2–3 weeks later, tumor-resected mice had tumors surgically removed, tumor-free control mice received a tumor resection sham surgery and tumor-bearing mice received a tumor induction. Behavior was tested during light or dark phase 2–3 weeks later, followed by tissue collection. The syringes and scalpel drawings have been obtained from free clipart through Microsoft PowerPoint, released under CC-BY-SA 4.0 license (the syringe is available at https://thumb9.shutterstock.com/photos/thumb_large/349072/142860406.jpg and the scalpel by Petit B https://creativecommons.org/licenses/by-sa/4.0, from Wikimedia Commons).

To verify that previously reported effects of tumor treatments on spleen mass were replicated^23^, that tumor growth/exposure was comparable between tumor-bearing and -resected mice, that tumors were not overwhelming to the point of cachexia, and to determine whether or not these measures were dependent on the time of day of tissue collection, respective tissue masses were compared among treatment groups. Tumors increased spleen mass relative to tumor-free controls, which was reversed by tumor resection and was independent of time of day (Supplementary Fig. 1A; *F*_*2,49*_ = 19.3; *p* < 0.0001; *post hoc* Bonferroni: *p* < 0.0001 in both cases; see Fig. 1 for experimental design and sample sizes). Final tumor mass of tumor-bearing mice were comparable (*p* > 0.05; Supplementary Fig. 1B) to masses of resected tumors, indicating that tumor-bearing and tumor-resected mice were exposed to comparable tumor burdens. Body weight change was comparable in all mice over time (*p* > 0.05; Supplementary Fig. 1C).

### Time of day modulated tumor-induced effects on behavior

Since the time of day in which tumor-induced behavioral changes are assessed varieus broadly and is often inconsistent with the rodents’ active phase, we tested the extent to which tumor-induced alterations in anxiety-like (open field) and hippocampal-dependent (spontaneous alternation) and -independent (novel object recognition) cognitive behavior are dependent on the time of day of behavioral assessment.

#### Open field test

**Figure 2:**
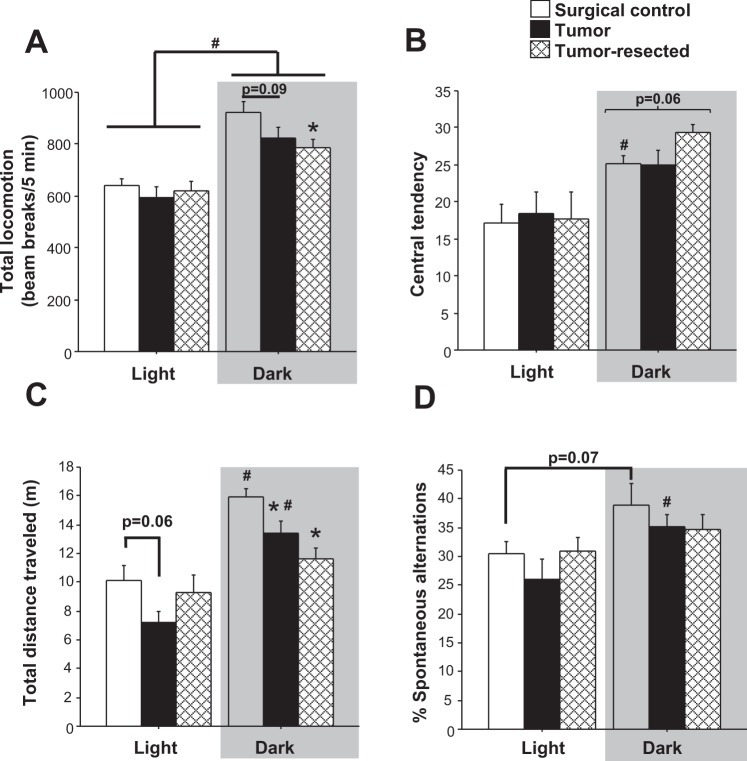
Effects of mammary tumors and complete tumor resection on anxiety-like, locomotor and cognitive behaviors. Mean ± SEM (**A**) total distance (beam breaks/5 minutes) traveled in open field test (**B**) central tendency in the open field test, (**C**) total distance (m) traveled in Y-maze spontaneous alteration test and (**D**) % of spontaneous alternations (n = 10–12/group); *p < 0.05 relative to control group at the same time point; ^#^*p* < 0.05 relative to the same treatment during the light phase by one-way and/or two-way ANOVA followed by Bonferroni *post hoc*.

All mice tested during the dark phase moved more in the open field than those tested during the light phase (Fig. 2A; *F*_*1,58*_ = 56.6, *p* < 0.0001); tumor treatments also tended to affect overall activity independently (*p* = 0.07). Indeed, tumor-bearing and tumor-resected mice moved less than controls, driven primarily by the mice tested during the dark phase (tumor main effect: *F*_*2,29*_ = 3.3, *p* < 0.05), when tumor-resected mice moved less than controls (*post hoc* Bonferroni: *p* < 0.05) and tumor-bearing mice tended to do the same (*p* = 0.09). Central tendency in the open field, indicative of anxiolytic behavior, increased for mice tested during the dark phase (Fig. 2B; *F*_*1,58*_ = 19.3, *p* < 0.0001), driven by the control (*U* = 18, *p* < 0.05) and tumor-resected (*U* = 25, *p* < 0.05) groups (relative to their light-phase counterparts), but not the tumor-bearing group (*p* > 0.05). During the dark phase, tumor-resected mice tended to spend more time in the center of the open field compared to both the controls and tumor-bearing mice (Fig. 2B; *F*_*2,29*_ = 3.2, *p* < 0.06).

### Spontaneous alternation test

Both time of day (Fig. 2C; *F*_*1,57*_ = 38.8, *p* < 0.0001) and tumor treatment (*F*_*2,57*_ = 5.1, *p* < 0.01) independently altered total distance traveled in the spontaneous alternation Y-maze, and their interaction approached statistical significance (*p* = 0.08). The significant effect of tumor treatment on total distance traveled was driven specifically by activity in the dark phase, in which both tumor-bearing and tumor-resected mice traveled less distance than control mice (*post hoc* Bonferroni: *p* < 0.05 in both cases). During the light phase, tumor mice tended to move less than controls (*p* = 0.06). Time-of-day differences in total distance traveled were driven by the control (*post hoc* Bonferroni: *p* < 0.001) and tumor-bearing groups (*post hoc* Bonferroni: *p* < 0.0005), whereas tumor-resected mice failed to demonstrate relatively elevated activity during the dark, active phase (*p* > 0.05). Mice also displayed higher percentages of correct spontaneous alternation during the dark phase compared to the light phase (Fig. 2D; *F*_*1,57*_ = 9.5, *p* < 0.005). This dark-phase improvement in spontaneous alternation was driven primarily by the tumor group (*post hoc* Bonferroni: *p* < 0.05) and a tendency by the control group (*p* = 0.07), whereas tumor-resected mice again failed to demonstrate a time-of-day difference in spontaneous alternation performance (*p* > 0.05).

#### Novel object recognition

**Figure 3:**
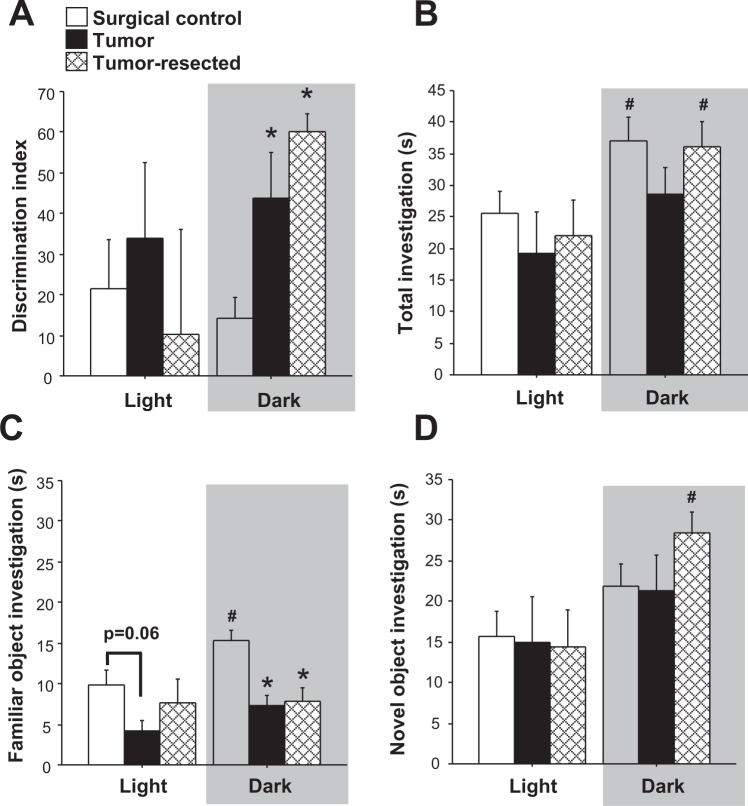
Effects of time of day on novel object recognition cognitive behavior. Mean ± SEM (**A**) discrimination index (**B**) total investigation (s) (**C**) familiar object investigation (s) and (**D**) novel object investigation (s) in surgical controls, tumor-bearing mice, and tumor-resected mice (n = 10–12/group); *p < 0.05 relative to control group at the same time point by one-way ANOVA; ^#^*p* < 0.05 relative to the same treatment during the light phase by two-way ANOVA.

Overall, the interaction between time of day and cancer treatment approached statistical significance for affecting the discrimination index for the novel object recognition task (Fig. 3A; *F*_*2,50*_ = 2.7, *p* = 0.08). Indeed, within mice tested during the dark phase, cancer treatment modulated the discrimination index (*F*_*2,20*_ = 110.1, *p* < 0.001) such that both tumor-bearing and tumor-resected mice spent 2-3-fold more time investigating the novel object versus the familiar object than surgical controls (*p* < 0.05 and *p* < 0.005, respectively). Specifically, total absolute investigation time was elevated in the dark phase relative to the light phase (Fig. 3B; *F*_*1,50*_ = 10.1, *p* < 0.005), driven by the control (*t*_18_ = 2.2, *p* < 0.05) and tumor-resected (*t*_17_ = 2.1, *p* < 0.05) groups, but not the tumor group (*p* > 0.05). Variability in the discrimination index was driven primarily by alterations in investigation of the familiar object (Fig. 3C), as opposed to the novel object (Fig. 3D). The familiar object was investigated more overall in the dark phase (*F*_*1,50*_ = 3.9, *p* ≤ 0.05), which was driven solely by the control mice (*t*_18_ = 2.4, *p* < 0.05) and absent in the tumor- and tumor-resected mice (*p* > 0.05 in both cases). Investigation of the novel object was also greater overall during the dark phase, but driven primarily by the tumor-resected group (*t*_17_ = 2.9, *p* < 0.05).

### Circulating inflammatory markers were modulated by time of day and tumor treatments

**Figure 4:**
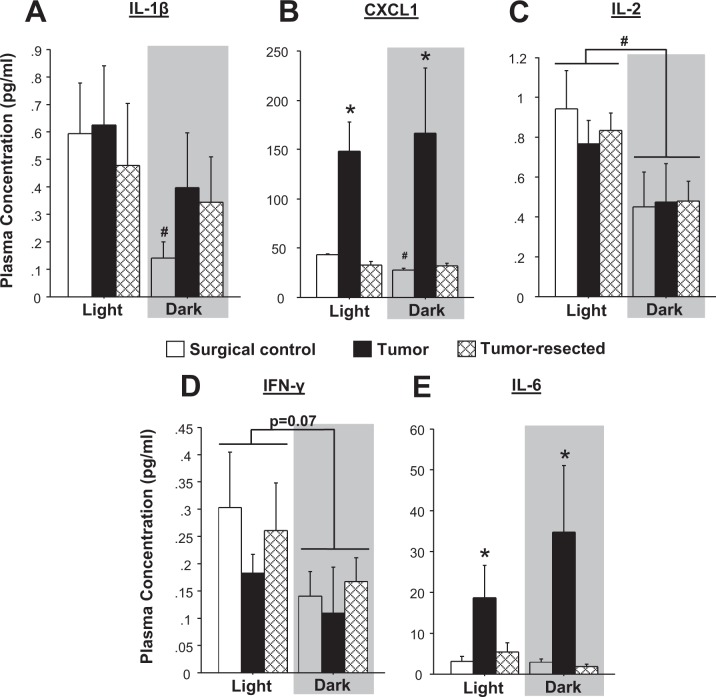
Circulating cytokines and chemokines are modulated by tumor group and time of day. Circulating plasma concentrations of (**A**) IL-β, (**B**) CXCL1, (**C**) IL-2, (**D**) IFN-γ, and (**E**) IL-6 (n = 10–12/group); *p < 0.05 between treatment groups at the same time point; ^#^*p* < 0.05 relative to the same treatment during the light phase by two-way ANOVA followed by Bonferroni *post hoc* or two-tailed Student’s *t*-test (day versus night).

To evaluate the extent to which tumor-induced alterations in systemic inflammatory signaling were time-of-day-dependent, plasma concentrations of a panel of inflammatory markers were quantified. Circulating levels of IL-1β and CXCL1 were higher in the light phase for tumor-free control mice (Fig. 4A-B**;** IL-1β: *t*_8_ = 2.3, *p* < 0.05; CXCL1: *t*_8_ = 10.9, *p* < 0.0001), but these time-of-day differences were lost in both the tumor-bearing and -resected mice. No statistically significant differences in circulating IL-1β were detected among treatment groups within either light cycle phase (Fig. 4A; *p* > 0.05). All mice displayed higher circulating IL-2 concentrations during the light phase (Fig. 4C; time-of-day effect: *F*_*1, 27*_ = 10.0, *p* < 0.005) relative to the dark phase; IL-2 levels were comparable among treatment groups in both phases. Time of day similarly tended to decrease the IFN-γ levels during the dark phase (Fig. 4D; *p* = 0.07). Tumors significantly increased overall CXCL1 and IL-6 at least three-fold (Fig. 4B–E; CXCL1: *F*_*2,27*_ = 17.7, *p* < 0.0001; IL-6: *F*_*2,27*_ = 9.6, *p* < 0.001) relative to both tumor-free and tumor-resected mice (*post hoc* Bonferroni: CXCL1: *p* < 0.0001 and *p* < 0.0001 respectively; IL-6: *p* < 0.005 and *p* < 0.001 respectively), regardless of time of day.

### Brain qPCR Arrays

**Figure 5:**
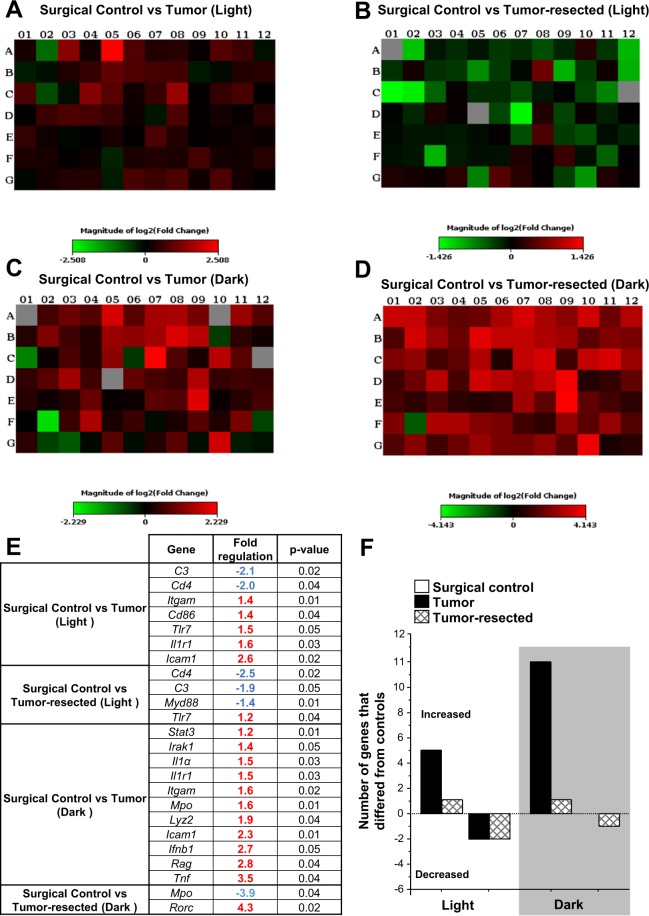
Transcriptional profiling of brain (frontal cortex + hippocampus) revealed time of day-dependent differential inflammatory gene expression in tumor-bearing and -resected mice. The log2 fold-change plate-layout heat maps of gene expression of (**A**) tumor-bearing and (**B**) -resected mice compared with surgical control during the light phase or dark phase (**C** and **D**), respectively). (**E**) Fold regulation and *p*-values of significant gene expression changes (by two-tailed Student’s *t*-test) from the PCR array relative to the respective control group (Light/Dark). Tumors induced upregulation of several immune-related genes, particularly during the dark phase (see Supplementary Table 1 and 2 for complete gene analyses). (**F**) Tumors and resection induced up- or downregulation of inflammatory genes in a time-of-day manner, as assessed by PCR array (n = 3–4/group).

To determine the influence of time of day on tumor or tumor-resection-induced neuroinflammatory signaling and to expand known neuroinflammatory changes associated with these tumor treatments^23^, the expression of 88 genes associated with innate and adaptive immunity were analyzed using PCR arrays. In general, the gene expression profile in the brain (frontal cortex and hippocampus combined) of the tumor-bearing and tumor-resected mice revealed differentially expressed genes compared to the surgical control mice in both phases of the light-dark cycle (Fig. 5A–D). Tumors induced differential expression (primarily increases) in more genes than tumor resection, which occurred during the dark phase twice as often as during the light phase (Fig. 5E-F). In contrast, most of the genes differentially expressed during the light phase were decreased in both the brains of tumor-bearing and tumor-resection mice compared with controls. See Supplementary Tables 1 and 2 for complete analyses.

### Tumor and tumor resection lost time-of-day differences in neuroimmune gene expression exhibited by controls

**Figure 6:**
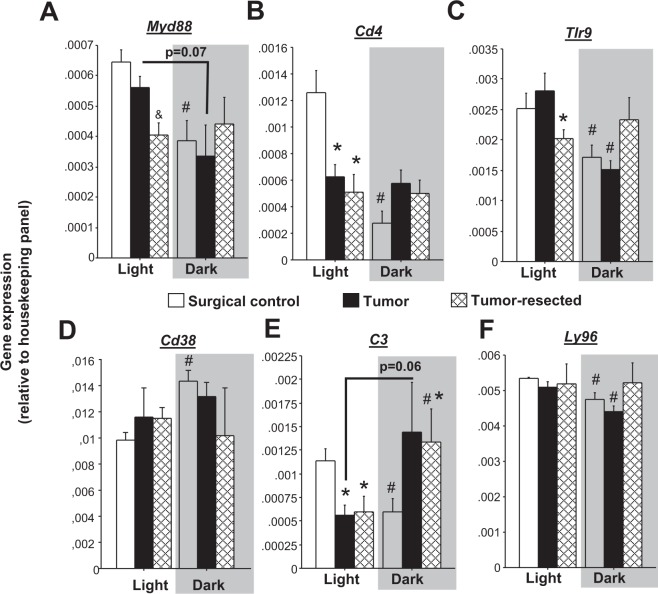
Effects of mammary tumors and tumor removal on nyctohemeral inflammatory gene expression in the brain. Mean ± SEM gene expression as assessed by RT^2^PCR arrays of brain (combined frontal cortex and hippocampus) (**A**) *Myd88*, (**B**) *Cd4*, (**C**) *Tlr9*, (**D**) *Cd38*, (**E**) *C3*, (**F**) *Ly96* (n = 3–4/group). *p < 0.05 relative to control group at the same time point; ^&^*p* < 0.05 relative to the other groups at the same time point by one-way ANOVA; ^#^*p* < 0.05 relative to the same treatment during the light phase by two-way ANOVA and/or two-tailed Student’s *t*-test (day versus night).

Temporal patterns of many immune/inflammatory transcripts in the brain have not previously been assessed. Here, time-of-day differences in combined hippocampal/frontal cortex samples of surgical control mice were observed for *Myd88, Cd4, Tlr9, Cd38, C3*, and *Ly96* (Fig. 6A–F**)**, such that mRNA expression was reduced in the dark phase relative to the light phase for all genes (except *Cd38*, which was increased in the dark phase). Tumors (Fig. 6B; *Cd4*, tendency in *Myd88*, *p* = 0.07) and tumor resection (Fig. 6A–D; *Myd88*, *Cd4*, *Tlr9, Cd38*) eliminated these time-of-day differences by reducing light-phase gene expression relative to controls and or increasing dark phase expression. In some cases, only tumor-resected mice lost this time-of-day difference (Fig. 6F; *Ly96*). Finally, complement component 3 (*C3*) gene expression was unique in that tumor- and tumor-resected mice displayed a time-of-day difference opposite to that of controls: elevated in the dark phase as opposed to elevated in the light phase (Fig. 6E).

### Select tumor treatment differences in neuroimmune gene expression were restricted to dark phase only

**Figure 7:**
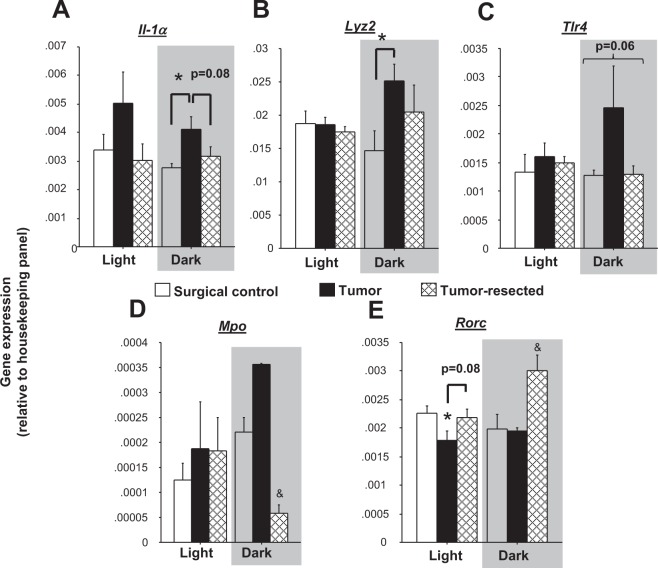
Dark phase-specific effects of mammary tumors and tumor removal on inflammatory gene expression in the brain. Mean ± SEM gene expression as assessed by RT^2^PCR array of brain (**A**) *Il1α* (**B**) *Lyz2*, (**C**) *Tlr4*, (**D**) *Mpo and* (**E**) *Rorc* (n = 3–4/group). *p < 0.05 relative to control group at the same time point; ^&^*p* < 0.05 relative to the other groups at the same time point by one-way ANOVA; ^#^*p* < 0.05 relative to the same treatment during the light phase by two-way ANOVA.

Tumor treatments tended to influence overall *Il1α* gene expression (Fig. 7A**;** *p* = 0.06), which was driven by samples from the dark phase, in which tumor-bearing mice displayed greater *Il1α* brain gene expression relative to surgical controls (tumor main effect: *F*_*2,7*_ = 5.1, *p* < 0.05; *post hoc* Bonferroni: *p* < 0.05). Similarly, tumors increased *Lyz2* (lysozyme 2) expression during the dark phase relative to controls (Fig. 7B**;** *F*_*2,7*_ = 3,1, *p* < 0.05). Tumors tended to increase *Tlr4* gene expression relative to controls and tumor-resected mice (Fig. 7C; *p* = 0.06) during the dark phase only. In contrast, tumor resection decreased dark-phase myeloperoxidase (*Mpo)* gene expression (Fig. 7D**;** *H* = 6, *p* < 0.05; *post hoc* Bonferroni/Dunn: *p* < 0.05) relative to the other groups. Tumor treatments (Fig. 7E**;** *F*_*2,16*_ = 7.8, *p* < 0.005) and the interaction between time of day and tumor treatments (*F*_*2,16*_ = 4.5, *p* < 0.05) both modulated retinoid-related orphan receptor C *(Rorc)* gene expression in the brain. Indeed, during the light phase, tumors decreased *Rorc* expression in the brain (*p* < 0.05), which tended to be reversed by tumor resection (*p* = 0.07), whereas during the dark phase, tumor resection increased *Rorc* relative to both controls and tumor-bearing mice (*p* < 0.05 in both cases). Furthermore, reductions in open field locomotion in all mice exposed to a tumor (combined tumor-bearing and -resected groups) were predicted by elevated brain *Il1α* and *Il1β* gene expression (Supplementary Fig. 2A,B; *p* < 0.05 in both cases).

### Tumors increased select neuroimmune gene expression during both the light and dark phases

Several immune/inflammatory genes in the brain increased in tumor-bearing rodents relative to controls and tumor-resected mice regardless of time of day: *Icam1* (Supplementary Fig. 3A; *F*_*2,16*_ = 22.3, *p* < 0.0001), *Ifnβ1* (Supplementary Fig. 3B; *F*_*2,11*_ = 8.2, *p* < 0.01), *Il1β* (Supplementary Fig. 3C; *F*_*2,15*_ = 4.4, *p* < 0.05), *Il1r1* (Supplementary Fig. 3D; *F*_*2,16*_ = 6.1, *p* < 0.05), *Itgam* (Supplementary Fig. 3E; *F*_*2,16*_ = 18.1, *p* < 0.0001), *Mapk1* (Supplementary Fig. 3F; *F*_*2,16*_ = 3.1, *p* = 0.07), *Tlr3* (Supplementary Fig. 3G; *F*_*2,16*_ = 3.6, *p* = 0.06), *Tlr7* (Supplementary Fig. 3H; *F*_*2,16*_ = 3.6, *p* ≤ 0.05, driven primarily by control vs. tumor). Moreover, reduced total investigation in all mice exposed to a tumor (combined tumor-bearing and -resected groups) was predicted by elevated brain *Ilr1*, *Icam1* and *Itgam* gene expression (Supplementary Fig. 4A–C; *p* < 0.05 in both cases), while reduced familiar object investigation, specifically, was predicted by elevated brain *Ilr1*, *Icam1* and *Mapk1* gene expression (Supplementary Fig. 4D–F**;** *p* < 0.05 in both cases).

## Discussion

In the present study, we demonstrate for the first time that tumors induce both time-of-day-dependent behavioral and neuroinflammatory changes. The relevance of the timing of both physiological and behavioral assessments is widely overlooked in biomedical research. Indeed, many studies report neither the timing of behavior nor tissue collections and/or assess behavior during the sleep period (light phase) in nocturnal rodents simply for the convenience of the diurnal, photophilic researchers. While, the significance of this timing is recognized in chronobiology and some behavioral neuroscience work^1,2^, this appreciation is not yet commonly acknowledged among cancer researchers. The present findings indicate that assessment timing significantly influences the results, and therefore interpretations, of mechanistic studies of psycho-oncology.

With respect to behavior, tumors induced changes in hippocampal-independent memory and lethargy, both of which were time-of-day-dependent. Indeed, these behavioral differences due to tumors were only elucidated because the study design compared behavior between mice tested during the dark (active) versus mice tested during the light (inactive) phases. In support of our hypothesis, for both these behavioral differences, assessments recorded specifically during the (arguably more clinically-relevant) active (dark) phase were altered by tumors. Tumor-induced fatigue-like behavior exhibited only during the dark phase was supported by decreased locomotion in both the open field test and spontaneous alternation maze, and was associated with increased peripheral and central inflammatory markers. Fatigue is a highly debilitating and prevalent symptom among breast cancer survivors^24,25^ and is consistent with previous preclinical^6–8^ and clinical studies^24^. Tumor removal, however, did not reverse tumor-induced reductions in locomotion, suggesting the potential for peripheral tumors to induce lasting changes in central pathways regulating behaviors such as fatigue. Similarly, we have previously demonstrated that tumor removal does not reverse tumor-induced anxiety-like behavior, but rather amplifies it^23^. In the present work, however, tumor-resected mice did not display increased anxiety-like behavior in a shorter version of the open field test (5 min versus 10 min in previous study^23^) during the dark phase. This most likely reflects the limitations of interpreting the initial 5 min of an open field test in this Balb/c strain, which is characterized by heightened anxiety-like behavior^26^.

Although previous studies using other rodent models indicate the potential for peripheral tumors to induce cognitive deficits^4,5,7^ (but see^10^), tumor-induced impairments were absent in the present cognitive assessments (novel object recognition and spontaneous alternation). In contrast to our prediction, the tumor and tumor-resected groups exhibited enhanced novel object recognition (i.e., discrimination index) during the dark phase. However, this calculation was primarily driven by reductions in familiar and increased total (familiar + novel) object investigation, rather than increased novel object investigation specifically. Notably and similar to the fatigue assessments, these cognitive differences were only apparent when testing occurred during the rodents’ active (dark) phase. Additional cognitive tasks are necessary to elucidate how tumors affect different facets of cognition, potentially in a time-of-day-dependent manner, in this and other models.

A growing body of literature suggests a causal role for peripheral tumor-associated inflammation in the induction of neuroinflammation and behavioral comorbidities^5,9,23,24,27^. Nevertheless, the specific underlying mechanisms and pathways by which inflammation spreads from the tumor to the CNS remain unknown. Expanding upon our previous work in this model^23^, in the present study, numerous additional inflammatory mediators in the brain were observed to increase after tumor induction independent of the time of day when the brain tissue was collected (e.g., *Icam1, Il1β, Il1r1, Ifnβ1, Itgam, Tlr7*). The intercellular adhesion molecule 1 (ICAM1), an important mediator of leukocyte trafficking, is upregulated in several types of solid tumors, including breast cancer^28^. Furthermore, increased circulating soluble ICAM1 (sICAM-1) concentrations have been associated with chemotherapy-induced fatigue and depressed mood^29^. The extent to which tumor-induced brain *Icam1* expression drives peripheral immune cell trafficking to brain, remain uninvestigated. Consistent with previous studies^23,30^, *Itgam* (also known as Cd11b) brain expression was elevated in tumor-bearing mice, suggesting a potential role for microglia in tumor-induced behavioral changes. Indeed, in other models of peripheral inflammation (e.g., stress), depletion of microglia alleviates inflammation-induced anxiety-like behavior^31^. Brain IL-1R1 is an important mediator for sickness behavior, monocyte recruitment to the CNS, and neurogenesis^32^. Signaling pathways downstream of IL-1R1 activation might have an important role in cancer-induced behavioral changes, given that cortical and hippocampal IL-1β increases are associated with behavioral outcomes in several other tumor rodent models^3,4,6,33^. Taken together, these peripheral tumor-induced genes in the brain are all associated with canonical pathogen-associated molecular patterns (PAMPs) or damage-associated molecular patterns (DAMPs) signaling pathways in microglia (i.e., *Itgam*), for example, through TLRs (e.g., TLR7), Myd88, and NF-κb, resulting in transcription of inflammatory signals (e.g., IL-1β, IFN, ICAM). In fact, TLR7 activation specifically increases IFNβ^34^, which are both similarly upregulated in the brain in the context of a peripheral immune challenge^35^. The causal impact of these increased transcripts in the observed behavioral changes requires further investigation. Nevertheless, in the current work, elevated brain *Il1α* and *Il1β* gene expression negatively predicted open field locomotion in all mice exposed to a tumor (combined tumor-bearing and -resected groups).

More relevant to time of day, some inflammatory mediators (*Myd88, Cd4, Tlr9, Cd38, C3, Ly96*) demonstrated nyctohemeral (day versus night) differences in control brain tissues that were abolished in tumor-bearing and/or tumor-resected mice. With the exception of *Cd38* (glycoprotein that regulates social behavior^36^), expression of all of these genes consistently decreased during the dark phase in control mice, consistent with the temporal pattern of inflammatory cytokines secreted by hippocampal microglia isolated from rats^22^. In stark contrast, this pattern was absent (*Myd88, Cd4, Tlr9, Ly96*) in tumor-bearing and/or –resected mice or reversed, in one case (*C3*). Of note, most of these transcripts also play a role in responses to PAMPs or DAMPs in the brain (*Myd88, Tlr9, Ly96, C3*). For example, PAMPs stimulate toll-like receptors (e.g., TLR9^37^), some of which function as protein complexes (e.g., TLR4 with Ly96), to transmit signaling pathways through the adaptor molecule, Myd88, and induce inflammation via NF-κB and other pathways. Alternatively, inflammation in the brain can be initiated through complement pathways (e.g., C3). Of note, all 13 TLRs are found on microglia in the brain with fewer TLRs expressed on neurons, astrocytes, and oligodendrocytes. Furthermore, PAMPs and DAMPs are prevalent in both pathogen and non-pathogen-driven neoplasms^38^, which is likely how tumors are disrupting the time of day patterns of these pathways in the brain.

Previous studies indicate that inflammatory signals secreted by microglial cells of the brain (primary sources of inflammatory signaling) exhibit circadian rhythmicity^22,39^, therefore, the abolishment of neuroinflammatory time-of-day differences by tumors or tumor resection may be indicative of microglial dysregulation. While microglia influence behavior both during homeostasis and disease^40^, the relevance of microglial rhythmicity in behavior is yet to be established. Circadian rhythms in peripheral immune cells, on the other hand, are more well-established, with phagocytotic activity of peritoneal macrophages elevated during the light phase and decreased in the dark phase^41^. Functionally, this synchronizes a greater pro-inflammatory response when mice are more likely to face a peripheral immune stimulant: during the dark (active) phase compared to the light (resting) phase^42^.

The circadian patterns of immune and inflammatory transcripts have been described in a number of mouse peripheral tissues. For example, TLR9 recognizes bacterial and viral DNA and *Tlr9* mRNA expression increases in spleen cells specifically during the dark phase in anticipation of immune responses against potential bacterial infections in the transition between inactive and active phases^43^. With respect to the genes of interest in the current study, the following genes are reported to exhibit rhythmicity in the mouse: *Myd88* (26-h rhythm in aorta^44^, but no rhythms in peritoneal-derived macrophages^45^); *Cd4* (28-h rhythm in liver^44^); *Cd38* (24-h rhythm in cerebellum and liver^44^); and *Ly96* (24-h rhythm in liver^44^). These rhythms of gene expression are likely cell- and tissue-specific, as a previous study demonstrated inconsistent rhythmicity and phases of gene expression among various tissues in multiple circadian microarray experiments^46^. Notably, there are limited descriptions of rhythmicity of many immune/inflammatory transcripts in the brain. *C3* has been previously reported to exhibit circadian oscillations (~28-h rhythm) in the circadian master clock (suprachiasmatic nucleus) of the hypothalamus^44,47^. A previous study of the prefrontal cortex in C57BL/6 mice examined genome-wide gene expression via microarray at 4 time points throughout a 24-h period did not find rhythmicity in any of the genes of interest in the current study^39^. However, the present study combined prefrontal cortex and hippocampal tissue, the strain of mice was different (BALB/c), and the sex of the mice in the previous study was unspecified, all factors that can influence rhythmicity^46^. To our knowledge there are no peer-reviewed RNAseq or microarray datasets examining circadian gene expression in the hippocampus. Future studies will distinguish whether the frontal cortex and/or hippocampus are driving the temporal patterns in gene expression profiled in the current study, whether the observed time-of-day differences are dependent on tumor type and location, and more frequent time sampling around the clock is necessary to conclusively assess circadian arrhythmicity of these neuroinflammatory signals.

Continuing the pattern observed from the behavioral analyses, that the most abundant differences among tumor treatments occurred during the dark phase, several additional genes varied among tumor treatments only during this time period. These included inflammatory mediators related to protection against bacterial infection (*Il1α*, *Tlr4*, *Lyz2, Mpo*), all of which increased in brains of tumor-bearing mice relative to tumor-free controls during the dark phase only. For *Mpo*, tumor resection also significantly reduced gene expression relative to tumor-bearing and control mice, suggesting a potential over-compensation of this transcript after the tumor was removed. Indeed, bacterial-related gene expression may be indicative of either tumor-induced DAMPs or gut permeability to bacteria^48^. Evidence within the cancer field suggests that chemotherapy-induced circulating proinflammatory cytokines may disrupt gut tight junction integrity, therefore, it stands to reason that tumors may alter gut permeability. Finally, RAR-related orphan receptor C (*Rorc*) brain mRNA displayed a unique pattern dependent upon tumor treatment, with tumors decreasing expression during the light phase and tumor resection increasing expression during the dark phase. Central *Rorc* regulates core clock gene expression (*Bmal*)^49^ and is also associated with inflammation and T-cell activation in the brain of an experimental stroke model^50^. Thus, the observed changes in brain *Rorc* may influence behavior through either immune and/or timing mechanisms. In summary, while these genes did not have time-of-day differences in expression in controls based on a 2-time point analysis, only the dark phase unmasked variation among the tumor treatments.

The pattern of induction of inflammatory mediators within the brain was similar to markers in the blood, since the genes (e.g., *Myd88*) or proteins (e.g., IL-2) that differed by time of day were all decreased during the dark phase. Additionally, tumors increased levels of other inflammatory markers (IL-6, CXCL1, *Icam1*, *Il1r1*) regardless of time of day in both blood and brain. In contrast to the inflammatory transcripts in the brain, none of the circulating cytokines assessed in the present study displayed a time of day by tumor treatment interaction. Based on the present data, time of day of assessment of some circulating cytokines may not be as large of a concern as it is for neuroinflammatory markers for this tumor model. The disconnection between the tumor-induced changes in brain and circulating inflammatory signaling may reflect temporal differences in clock gene activity between the brain and peripheral immune cells^22,45^. Indeed, while numerous circulating immunological mediators are regulated by central and peripheral clocks^51,52^, additional resolution (i.e., sampling points) is likely necessary to demonstrate this. Given that proinflammatory cytokines are involved in chronic pain development^53^, it is important to highlight that all mice received surgery (tumor resection or sham resection surgeries [controls] and/or tumor induction surgery) 2–3 weeks prior to tissue collection, indicating that wound healing or surgical pain do not explain differences among treatment groups.

Taken together, this work indicates that differences among tumor treatments are unmasked by testing during the active phase for both behavior and brain inflammatory measures, whereas the circulating inflammatory changes assessed were independent of time of day. The potential causal role of the neuroinflammatory changes occurring among tumor treatments during the dark phase on the coincident behavioral changes require further investigation, however significant correlations between these measures were observed for fatigue (locomotion and investigation) and cognitive (total novel object and familiar object investigation) behaviors. The overall implication of these data is that not only are assessments during appropriate, waking hours germane to behavioral results and their interpretation, but also to neurobiological results. Time-of-day variation in neurobiology may be indicative of clock-mediated circadian rhythms, or simply indicative of neurobiological variation based on rest-activity cycles. Indeed, chronobiology may be one of the most untapped phenomena to impact modern medicine^54^. Debilitating cancer-associated behavioral comorbidities are repeatedly associated with physiological biomarkers that are rhythmic^55^. Thus, understanding how the timing of biobehavioral research affects results and their interpretation will inform and optimize the timing of treatment and biomarker assessments, to improve long-term quality of life and survival in cancer patients.

## Methods

### Animals

Nulliparous female 8- to 9-week old Balb/c mice (Charles River, Wilmington, MA; see Fig. 1 for sample sizes) were housed 5/cage and acclimated to the temperature-controlled (22 ± 1 °C) vivarium for 1 week under a 14:10 light:dark cycle (lights off at 016:00 h). Rodent chow (Harlan 7912) and water were available *ad libitum* throughout the study and cotton nestlets and plastic huts were provided for nesting. Prior to and after tumor induction, mice were acclimated to handling twice/week. All animal experiments were approved by the Ohio State University Institutional Animal Care and Use Committees and carried out in accordance with the National Institutes of Health Guide for the Care and Use of Laboratory Animals^56^. All efforts were made to minimize animal suffering and to reduce the number of mice used.

### Cells

The murine mammary non-metastatic 67NR cancer cell line, originating from a spontaneous mammary adenocarcinoma in a Balb/c mouse^57,58^, was generously provided by Drs. Fred Miller and Lisa Polin at Karmanos Cancer Institute. Cells were grown in DMEM with 10% FBS, 2 mM _L_-glutamine, 1 mM non-essential amino acids at 37 °C with 5% CO_2_.

### Tumor Induction

Non-metastatic, syngeneic, orthotopic mammary tumors were induced, and then some were resected, as previously described^23^. Briefly, under anesthetization (isoflurane vapors), a 5 mm subcutaneous incision was made medial to the 4^th^ nipple and 5 × 10^6^ cells (in matrigel) were injected into the associated mammary fat pad and the incision was closed with a wound clip. Ear notches were placed for individual identification purposes at this time. To control for the effects of surgeries (i.e., tissue repair, anesthesia), tumor-free control mice received a tumor resection sham surgery (n = 20, 10 for each time of day) at the same time as tumor resection (Fig. 1). Mice slated for the tumor resection group were inoculated with tumor cells 2.5 weeks prior to the other groups such that the timing of their tumor resection surgeries corresponded to the time of tumor induction of the other groups (Fig. 1). This allowed for simultaneous behavioral and physiological assessments in all groups and an equal duration of tumor exposure (2 weeks) between tumor and tumor-resected mice for the start of behavioral analyses. Body mass and tumor dimensions were measured twice/week. The longest diameter of the tumor (A) and the perpendicular diameter (B) were used to estimate tumor volume by the formula: V = A × B^2^/2 mm^3^. Mice in the tumor group with final tumors <4 (n = 3) or >16 (n = 6) mm dia (~10% body mass) were euthanized and removed from the study early.

### Tumor resection

A modified radical mastectomy procedure was used to completely remove the tumor in the mice of the “survivor” group as previously described^23^. Briefly, these mice (n = 28, 14 for each time of day) were anesthetized (isoflurane) and tumors (once reached ~13 mm long) with intact capsules were surgically removed along with mammary tissue, fat, and inguinal lymph nodes where necessary and the skin was closed with wound clips. Sham resection surgeries included probing and exposing these healthy tissues in the surgical control group (n = 20). Buprenorphine (0.05 mg/kg; s.c.) was administered immediately after surgery and again every 6–12 h for up to 72 h after surgery and wound clips were removed after 7 d. Complete tumor resections were verified at necropsy, mice with recurring tumors (n = 5) were removed from the study and were not included in the final sample sizes.

### Behavior

Three behavioral tests (open field, spontaneous alternation, novel object recognition) were assessed simultaneously in all mice, which commenced 2 weeks after tumor induction (tumor groups) and 2–3 weeks after tumor resection (tumor-resected/surgical control group), given that tumor resection surgeries occurred over a 5-day period based on individual tumor growth rate. Behavioral testing consistently occurred at 07:00 h in half of the mice (light phase groups) and at 17:00 h in the other half (dark phase groups; n = 10–12/group/time) over four consecutive days. Each behavior test was separated by 24 h (open field took 2 days to complete all mice). Behavioral assessments during the light phase were conducted under standard overhead white lighting, whereas darkroom-grade dim red light and an infrared-sensitive camera were used for testing during the dark phase.

#### Open field test

Automated equipment was used to assess total locomotor activity and anxiety-like behavior (avoidance of the center 4 × 4 in area) in 16 × 16 in plastic arenas (San Diego Instruments, San Diego, CA, USA). Sixteen photobeams across two dimensions recorded spatial movement of each mouse over 5 min. A loose layer of bedding was added and the arenas were cleaned with 10% bleach between mice. Standard overhead white light was used during the light phase, while no light or darkroom-grade dim red light was used during the dark phase.

#### Spontaneous alternation

This spatial working memory test was performed based on a modified version of the procedure developed by Lalonde^59^. A Y-maze, consisting of 3 equal-length gray acrylic arms (40 × 8 × 15 cm l × w × h), was used. Mice were placed initially in the center and allowed to explore the entire maze for 3 min. This test is based on the natural tendency of rodents to methodically explore relatively novel arms rather than the most recently explored arms^59^. The series of arm entries was recorded using an overhead camera and tracked using ANY-Maze video tracking software (Stoelting Co., San Diego, CA, USA). An alternation was defined as successive entries into each of the three arms, in any order. Percent spontaneous alternation was calculated as the ratio of successful alternations over total possible alternations (defined as the total number of arm entries minus two), multiplied by 100^60^.

#### Novel object recognition

This non-spatial cognitive task is mediated by the temporal cortex and is based on rodents’ preference to investigate novel versus familiar objects. This task was performed as previously described^3^. Briefly, after completing the spontaneous alternation test, mice were allowed to investigate 2 identical objects (upright 50 ml conical tubes filled with sand) for 10 min in a clean cage, after which they were returned to their home cage. Three hours later, mice were returned to the clean cage, but one familiar object was replaced with a novel object (hockey puck). Investigative behavior was scored for 5 min. All objects were rinsed with 70% ethanol between trials. Videos were scored using Etholog v. 2.2 freeware (Sao Paulo, Brazil). Investigation was defined as object-directed behavior with the nose <1 cm from an object and vibrissae moving. A novel object discrimination index (DI) was calculated: (Time spent investigating novel object – Time spent investigating familiar object)/(Total time spent investigating either object) × 100.

#### Tissue collection

Mice from all groups were killed one day (24 h) after the novel object recognition test during the light phase (0600–1100 h) or the dark phase (1600–2100 h), respectively. Our previous study evaluated the extent to which tumor treatment influences neuroinflammatory and sickness behavior responses to an immune challenge (lipopolysaccharide - LPS) in mice. This study indicate the tumor treatment attenuates LPS-induced lethargy during the dark phase^61^. Based on this finding, our *a priori hypothesis* was that tumor treatment-induced neuroinflammation would be higher at the beginning of the inactive phase (light) relative to the active phase (dark)^21,62^. Four mice/group/time were saline perfused following deep CO_2_ asphyxiation, and brain regions relevant to the behaviors examined (hippocampus, frontal cortex) were immediately dissected out and frozen in RNA Later preservative for later neuroinflammatory gene expression assessment. Mice not perfused were killed via rapid decapitation and trunk blood was collected through heparin lined blood collecting tubes. Tumors and spleens from all animals were removed aseptically and weighed.

### Plasma inflammatory marker ELISAs

Concentrations of multiple circulating cytokines and chemokines were measured using a customized multiplex immunoassay (cat. #: K15069L-2, lot #: 252145, Meso Scale Discovery, Rockville, MD, USA). Plasma samples were diluted 2x with dilution buffer and run in duplicate according to the manufacturer’s protocol using a QuickPlex SQ 120 instrument (Meso Scale Discovery, Rockville, MD, USA). Detection thresholds for each cytokine or chemokine were the following: CXCL1: 1.37 pg/ml; IL-6: 5.99 pg/ml; IFN-γ: 0.17 pg/ml; IL-1β: 1.63 pg/ml; IL-2: 0.75 pg/ml; IL-10: 11.1 pg/ml; TNFα: 5.94 pg/ml. Values below these limits were extrapolated based on fit to the standard curve, and a concentration of 0 pg/ml was used when signals were indistinguishable from standards containing 0 pg/ml of the corresponding cytokine. Values for IL-10 and TNFα were not included in the analysis due to a majority of samples containing undetectable signals for these cytokines. Intra-assay variations were <15% across the entire plate for each signal analyzed.

### Quantitative RT-PCR Arrays

Total RNA was extracted from the brain hippocampus and frontal cortex of a subset of mice (n = 4/group/time of day) using Qiagen RNeasy mini kits (CA, USA). RNA concentrations were measured and 260/280 ratios were determined to be 2.0–2.1 (NanoDrop, DE, USA). A customized, validated RT^2^ Profiler PCR array (Qiagen, Germantown, MD, USA; Cat. No. CAPM13840-PAMM-052Z), designed to measure 88 mouse genes associated with the innate and adaptive immune response (84 standard genes + *Cd68*, *Cx3cl1*, *Cd38*, *Cx3cr1*), was run (QuantStudio™ 5 System, Applied Biosystems, Foster City, CA, USA) according to the manufacturer’s instructions using pooled RNA from hippocampal and frontal cortex tissue. Briefly, 500 ng of isolated RNA was synthesized to cDNA, SYBR Green qPCR Master Mix was used, and gene expression was normalized using the geometric mean of a panel of housekeeping genes including Beta actin (*Actb*), Beta-2 microglobulin (*B2m*), glyceraldehyde 3-phosphate dehydrogenase (*Gapdh*), Beta-glucuronidase (*Gusb*), and Heat shock protein HSP 90-beta (*Hsp90ab1*). Relative gene expression of individual samples was calculated by the comparative C_T_ method (2^−ΔCT^). Heatmaps and the Supplementary Tables 1 and 2 were generated using the QIAGEN GeneGlobe Data Analysis Center (https://dataanalysis.qiagen.com/pcr/arrayanalysis). Two mice met criteria (±2SD of mean; 1 tumor-bearing and 1 –resected mouse from the dark phase) for outliers and were removed from the analyses.

### Statistical analysis

All statistical comparisons were analyzed using the software Statview version 5.0.1 software (Scientific Computing, Cary, NC, USA) and consisted of 2-way ANOVAs (with tumor treatment and time of the day as variables) followed by *post-hoc* Bonferroni/Dunn’s corrections or Student’s *t*-tests based on *a priori* hypothesis when variance was normal. The *a priori* hypothesis was that tumor treatment alters behavior and physiology primarily during one phase of the day (active/dark phase for behavior, inactive/light phase for immune activity). Nonparametric Kruskal-Wallis or Mann-Whitney U tests were used when variance was not normally distributed. Pearson’s correlations were used to relate various variables between cytokines gene expression and behavior. All data are presented as mean ± standard error of the mean (SEM) and were considered to be statistically significant when *p* < 0.05.

## Supplementary information

Supplementary Information
