# Supplementary material for: Cancer and cancer survival modulates brain and behavior in a time-of-day-dependent manner in mice
Source: Sci Rep. 2019 Apr 24;9:6497. doi: 10.1038/s41598-019-42880-w (PMC6482139; doi:10.1038/s41598-019-42880-w)
Supplement: Supplementary file 1 — Supplementary Information [file 41598_2019_42880_MOESM1_ESM.docx]

**SUPPLEMENTARY INFORMATION**

**Cancer and cancer survival modulates brain and behavior in a time-of-day-dependent manner in mice**

Jessica C. Santos, Savannah R. Bever, Kyle A. Sullivan, Leah M. Pyter

**SUPPLEMENTARY TABLE 1**

| **Position** | **Gene Description** | **Gene Symbol** | **Up-Down Regulation (compared to the respective Surgical Control [Light/Dark])** | | | | | | | |
| --- | --- | --- | --- | --- | --- | --- | --- | --- | --- | --- |
|  |  |  | **Tumor Light** | | **Tumor-resected Light** | | **Tumor Dark** | | **Tumor-resected Dark** | |
|  |  |  | **Fold**  **Regulation** | **p-value** | **Fold Regulation** | **p-value** | **Fold Regulation** | **p-value** | **Fold Regulation** | **p-value** |
| A01 | Serum amyloid P-component | Apcs | 1.18 | 0.90 | -1.21 | 0.72 | 1.88 | 0.35 | 8.63 | 0.29 |
| A02 | Complement component 3 | C3 | -2.09 | **0.02** | -1.91 | **0.05** | 2.58 | 0.32 | 2.26 | 0.05 |
| A03 | Complement component 5a receptor 1 | C5ar1 | 2.56 | 0.12 | -1.09 | 0.65 | 2.03 | 0.20 | 4.24 | 0.28 |
| A04 | Caspase 1 | Casp1 | 1.14 | 0.61 | -1.18 | 0.61 | 1.48 | 0.36 | 2.66 | 0.29 |
| A05 | Chemokine (C-C motif) ligand 12 | Ccl12 | 5.69 | 0.06 | -1.08 | 0.95 | 3.43 | 0.24 | 3.07 | 0.30 |
| A06 | Chemokine (C-C motif) ligand 5 | Ccl5 | 1.72 | 0.27 | -1.24 | 0.53 | 2.11 | 0.26 | 8.69 | 0.29 |
| A07 | Chemokine (C-C motif) receptor 4 | Ccr4 | 1.26 | 0.72 | -1.18 | 0.79 | 2.38 | 0.26 | 9.86 | 0.29 |
| A08 | Chemokine (C-C motif) receptor 5 | Ccr5 | 1.30 | 0.21 | -1.34 | 0.60 | 3.36 | 0.35 | 8.14 | 0.23 |
| A09 | Chemokine (C-C motif) receptor 6 | Ccr6 | 1.05 | 0.58 | -1.15 | 0.70 | 2.23 | 0.11 | 5.32 | 0.25 |
| A10 | Chemokine (C-C motif) receptor 8 | Ccr8 | 1.78 | 0.27 | 1.15 | 0.62 | 1.88 | 0.35 | 9.25 | 0.29 |
| A11 | CD14 antigen | Cd14 | 1.51 | 0.12 | -1.22 | 0.75 | 2.56 | 0.18 | 3.38 | 0.27 |
| A12 | CD4 antigen | Cd4 | -2.02 | **0.04** | -2.47 | **0.02** | 2.11 | 0.16 | 1.83 | 0.28 |
| B01 | CD40 antigen | Cd40 | -1.21 | 0.39 | -1.21 | 0.70 | 1.28 | 0.53 | 2.44 | 0.31 |
| B02 | CD40 ligand | Cd40lg | -1.11 | 0.88 | 1.07 | 0.88 | 1.81 | 0.31 | 9.09 | 0.29 |
| B03 | CD80 antigen | Cd80 | 1.23 | 0.26 | -1.19 | 0.87 | 1.45 | 0.43 | 5.52 | 0.28 |
| B04 | CD86 antigen | Cd86 | 1.42 | **0.04** | -1.25 | 0.50 | 1.21 | 0.31 | 3.26 | 0.28 |
| B05 | CD8 antigen, alpha chain | Cd8a | 1.93 | 0.34 | -1.72 | 0.33 | 2.26 | 0.25 | 11.50 | 0.29 |
| B06 | C-reactive protein, pentraxin-related | Crp | 1.70 | 0.39 | -1.25 | 0.62 | 2.02 | 0.29 | 7.58 | 0.29 |
| B07 | Colony stimulating factor 2 (granulocyte-macrophage) | Csf2 | 1.59 | 0.42 | -1.02 | 0.89 | 2.32 | 0.30 | 7.79 | 0.29 |
| B08 | Chemokine (C-X-C motif) ligand 10 | Cxcl10 | 1.57 | 0.31 | 1.53 | 0.35 | 3.88 | 0.17 | 7.32 | 0.28 |
| B09 | Chemokine (C-X-C motif) receptor 3 | Cxcr3 | -1.18 | 0.98 | -2.00 | 0.26 | 2.85 | 0.09 | 5.76 | 0.29 |
| B10 | DEAD (Asp-Glu-Ala-Asp) box polypeptide 58 | Ddx58 | -1.06 | 0.87 | -1.25 | 0.72 | -1.39 | 0.86 | 2.76 | 0.24 |
| B11 | Fas ligand (TNF superfamily, member 6) | Fasl | 1.26 | 0.88 | 1.07 | 0.99 | 1.17 | 0.48 | 3.79 | 0.29 |
| B12 | Forkhead box P3 | Foxp3 | 1.32 | 0.69 | -1.98 | 0.21 | -1.07 | 0.61 | 3.21 | 0.29 |
| C01 | GATA binding protein 3 | Gata3 | 1.66 | 0.41 | -2.69 | 0.60 | -2.56 | 0.44 | 3.57 | 0.36 |
| C02 | Histocompatibility 2, Q region locus 10 | H2-Q10 | -1.65 | 0.12 | -2.52 | 0.11 | 1.12 | 0.91 | 5.48 | 0.29 |
| C03 | Histocompatibility 2, T region locus 23 | H2-T23 | -1.09 | 0.58 | -1.47 | 0.10 | 1.53 | 0.31 | 2.04 | 0.29 |
| C04 | Intercellular adhesion molecule 1 | Icam1 | 2.57 | **0.02** | 1.06 | 0.86 | 2.31 | **0.004** | 1.03 | 0.28 |
| C05 | Interferon alpha 2 | Ifna2 | 1.80 | 0.21 | -1.17 | 0.72 | 2.37 | 0.20 | 7.36 | 0.29 |
| C06 | Interferon (alpha and beta) receptor 1 | Ifnar1 | 1.12 | 0.47 | -1.17 | 0.58 | -1.35 | 0.71 | 1.46 | 0.23 |
| C07 | Interferon beta 1, fibroblast | Ifnb1 | 2.90 | 0.45 | -1.33 | 0.59 | 3.91 | **0.05** | -2.24 | 0.29 |
| C08 | Interferon gamma | Ifng | 2.80 | 0.16 | -1.09 | 0.80 | 1.36 | 0.39 | 9.52 | 0.29 |
| C09 | Interferon gamma receptor 1 | Ifngr1 | 1.03 | 0.85 | -1.00 | 0.87 | 1.19 | 0.67 | 2.04 | 0.20 |
| C10 | Interleukin 10 | Il10 | 1.41 | 0.50 | -1.29 | 0.63 | 2.75 | 0.23 | 8.15 | 0.29 |
| C11 | Interleukin 13 | Il13 | 1.61 | 0.30 | -1.66 | 0.58 | 1.74 | 0.35 | 12.02 | 0.29 |
| C12 | Interleukin 17A | Il17a | 1.02 | 0.80 | -1.21 | 0.72 | 1.88 | 0.35 | 5.05 | 0.29 |
| D01 | Interleukin 18 | Il18 | 1.03 | 0.91 | -1.01 | 1.00 | 1.31 | 0.40 | 1.88 | 0.31 |
| D02 | Interleukin 1 alpha | Il1a | 1.46 | 0.26 | -1.16 | 0.67 | 1.48 | **0.03** | 1.01 | 0.29 |
| D03 | Interleukin 1 beta | Il1b | 1.67 | 0.23 | 1.11 | 0.96 | 2.70 | 0.14 | 1.02 | 0.29 |
| D04 | Interleukin 1 receptor, type I | Il1r1 | 1.62 | **0.03** | 1.03 | 0.77 | 1.48 | **0.03** | 1.03 | 0.27 |
| D05 | Interleukin 2 | Il2 | 1.42 | 0.51 | -1.21 | 0.72 | 1.88 | 0.35 | 9.55 | 0.29 |
| D06 | Interleukin 23, alpha subunit p19 | Il23a | 1.10 | 0.55 | -1.31 | 0.28 | 1.85 | 0.10 | 8.02 | 0.29 |
| D07 | Interleukin 4 | Il4 | -1.14 | 0.68 | -2.61 | **0.04** | 1.68 | 0.63 | 6.95 | 0.29 |
| D08 | Interleukin 5 | Il5 | 1.68 | 0.18 | 1.13 | 0.67 | 1.22 | 0.99 | 7.75 | 0.29 |
| D09 | Interleukin 6 | Il6 | 1.03 | 0.60 | -1.30 | 0.66 | 2.50 | 0.22 | 13.41 | 0.28 |
| D10 | Interleukin-1 receptor-associated kinase 1 | Irak1 | 1.15 | 0.34 | 1.04 | 0.71 | 1.38 | **0.05** | 1.53 | 0.15 |
| D11 | Interferon regulatory factor 3 | Irf3 | 1.04 | 0.95 | -1.12 | 0.76 | 1.34 | 0.42 | 1.60 | 0.34 |
| D12 | Interferon regulatory factor 7 | Irf7 | 1.35 | 0.19 | 1.00 | 0.87 | 1.38 | 0.34 | 2.94 | 0.28 |
| E01 | Integrin alpha M | Itgam | 1.40 | **0.004** | 1.03 | 0.79 | 1.62 | **0.02** | 1.05 | 0.27 |
| E02 | Janus kinase 2 | Jak2 | 1.12 | 0.52 | -1.08 | 0.53 | 1.02 | 0.77 | 1.54 | 0.23 |
| E03 | Lymphocyte antigen 96 | Ly96 | -1.04 | 0.57 | -1.07 | 0.82 | -1.07 | 0.37 | 1.10 | 0.29 |
| E04 | Lysozyme 2 | Lyz2 | -1.01 | 0.95 | -1.08 | 0.42 | 1.71 | **0.04** | 1.17 | 0.28 |
| E05 | Mitogen-activated protein kinase 1 | Mapk1 | 1.18 | 0.08 | -1.05 | 0.70 | 1.19 | 0.06 | 1.12 | 0.19 |
| E06 | Mitogen-activated protein kinase 8 | Mapk8 | 1.03 | 0.85 | 1.02 | 0.92 | 1.06 | 0.65 | 1.63 | 0.24 |
| E07 | Mannose-binding lectin (protein C) 2 | Mbl2 | 1.65 | 0.36 | -1.35 | 0.57 | 1.53 | 0.50 | 5.36 | 0.29 |
| E08 | Myeloperoxidase | Mpo | 1.26 | 0.53 | 1.33 | 0.47 | 1.60 | **0.01** | -3.92 | **0.04** |
| E09 | Myxovirus (influenza virus) resistance 1 | Mx1 | 1.02 | 0.77 | -1.25 | 0.38 | 4.25 | 0.16 | 19.28 | 0.29 |
| E10 | Myeloid differentiation primary response gene 88 | Myd88 | -1.15 | 0.90 | -1.60 | **0.01** | -1.15 | 0.56 | 1.15 | 0.26 |
| E11 | Nuclear factor of kappa light polypeptide gene enhancer in B-cells 1, p105 | Nfkb1 | 1.12 | 0.53 | -1.05 | 0.84 | 1.19 | 0.21 | 1.96 | 0.28 |
| E12 | Nuclear factor of kappa light polypeptide gene enhancer in B-cells inhibitor, alpha | Nfkbia | 1.07 | 0.35 | -1.14 | 0.48 | 1.40 | 0.31 | 1.53 | 0.35 |
| F01 | NLR family. pyrin domain containing 3 | Nlrp3 | 1.20 | 0.19 | -1.05 | 0.84 | 1.08 | 0.79 | 4.38 | 0.29 |
| F02 | Nucleotide-binding oligomerization domain containing 1 | Nod1 | 1.17 | 0.19 | -1.08 | 0.73 | -5.42 | 0.44 | -3.88 | 0.44 |
| F03 | Nucleotide-binding oligomerization domain containing 2 | Nod2 | 1.03 | 0.83 | -2.01 | 0.15 | 1.44 | 0.24 | 7.08 | 0.29 |
| F04 | Recombination activating gene 1 | Rag1 | 1.08 | 0.97 | -1.11 | 0.72 | 2.76 | **0.04** | 6.59 | 0.29 |
| F05 | RAR-related orphan receptor gamma | Rorc | -1.26 | 0.07 | -1.05 | 0.52 | 1.00 | 0.55 | 4.32 | **0.02** |
| F06 | Solute carrier family 11 (proton-coupled divalent metal ion transporters), member 1 | Slc11a1 | 1.19 | 0.43 | -1.06 | 0.73 | 1.40 | **0.004** | 3.84 | 0.28 |
| F07 | Signal transducer and activator of transcription 1 | Stat1 | 1.27 | 0.17 | 1.13 | 0.57 | -1.07 | 0.96 | 2.04 | 0.24 |
| F08 | Signal transducer and activator of transcription 3 | Stat3 | 1.19 | 0.08 | 1.01 | 0.83 | 1.17 | **0.01** | 1.66 | 0.17 |
| F09 | Signal transducer and activator of transcription 4 | Stat4 | 1.59 | 0.38 | 1.25 | 0.85 | 1.53 | 0.07 | 4.68 | 0.28 |
| F10 | Signal transducer and activator of transcription 6 | Stat6 | 1.14 | 0.55 | -1.06 | 0.99 | 1.21 | 0.35 | 2.81 | 0.28 |
| F11 | T-box 21 | Tbx21 | 1.13 | 0.78 | -1.45 | 0.32 | 2.14 | 0.32 | 5.71 | 0.28 |
| F12 | Toll-like receptor adaptor molecule 1 | Ticam1 | 1.29 | 0.58 | 1.01 | 0.84 | -1.48 | 0.36 | 2.48 | 0.23 |
| G01 | Toll-like receptor 1 | Tlr1 | -1.04 | 0.72 | 1.12 | 0.57 | 1.21 | 0.48 | 2.28 | 0.30 |
| G02 | Toll-like receptor 2 | Tlr2 | 1.15 | 0.46 | 1.08 | 0.64 | -1.50 | 0.62 | 4.55 | 0.28 |
| G03 | Toll-like receptor 3 | Tlr3 | 1.24 | 0.13 | 1.05 | 0.77 | 1.29 | 0.66 | -1.12 | 0.28 |
| G04 | Toll-like receptor 4 | Tlr4 | 1.27 | 0.50 | 1.17 | 0.72 | 1.52 | 0.06 | 1.02 | 0.29 |
| G05 | Toll-like receptor 5 | Tlr5 | -1.20 | 0.62 | -1.65 | 0.16 | 1.86 | 0.26 | 6.09 | 0.29 |
| G06 | Toll-like receptor 6 | Tlr6 | 1.64 | 0.15 | 1.39 | 0.46 | -1.09 | 0.91 | 3.68 | 0.28 |
| G07 | Toll-like receptor 7 | Tlr7 | 1.49 | **0.02** | 1.17 | **0.04** | 3.12 | 0.34 | 1.20 | 0.09 |
| G08 | Toll-like receptor 8 | Tlr8 | 1.67 | 0.41 | 1.02 | 0.73 | 1.57 | 0.13 | 4.66 | 0.29 |
| G09 | Toll-like receptor 9 | Tlr9 | 1.12 | 0.48 | -1.27 | 0.09 | -1.13 | 0.97 | -1.12 | 0.27 |
| G10 | Tumor necrosis factor | Tnf | 1.68 | 0.81 | -1.79 | 0.32 | 3.51 | **0.04** | 15.01 | 0.28 |
| G11 | Tnf receptor-associated factor 6 | Traf6 | 1.15 | 0.31 | 1.12 | 0.39 | -1.19 | 0.83 | 1.29 | 0.23 |
| G12 | Tyrosine kinase 2 | Tyk2 | 1.03 | 0.88 | 1.03 | 0.93 | -1.08 | 0.99 | 1.60 | 0.29 |
| H01 | Actin, beta | Actb | -1.14 | 0.17 | 1.02 | 0.82 | -1.13 | 0.37 | -1.34 | 0.25 |
| H02 | Beta-2 microglobulin | B2m | -1.01 | 1.00 | 1.00 | 0.85 | 1.53 | 0.32 | 1.58 | 0.33 |
| H03 | Glyceraldehyde-3-phosphate dehydrogenase | Gapdh | 1.09 | 0.13 | -1.04 | 0.26 | 1.11 | **0.04** | 1.31 | 0.23 |
| H04 | Glucuronidase, beta | Gusb | 1.05 | 0.70 | -1.22 | 0.21 | 1.30 | 0.33 | 1.66 | 0.31 |
| H05 | Heat shock protein 90 alpha (cytosolic), class B member 1 | Hsp90ab1 | 1.05 | 0.35 | 1.02 | 0.69 | 1.01 | 0.83 | 1.01 | 0.88 |
| H06 | Mouse Genomic DNA Contamination | MGDC | 1.20 | 0.84 | -1.12 | 0.84 | 1.88 | 0.35 | 7.55 | 0.29 |
| H07 | Reverse Transcription Control | RTC | 1.06 | 0.89 | -1.04 | 0.82 | 1.77 | 0.35 | 2.62 | 0.30 |
| H08 | CD68 antigen | Cd68 | 1.24 | 0.10 | 1.17 | 0.25 | 1.54 | 0.26 | 1.78 | 0.31 |
| H09 | Chemokine (C-X3-C motif) ligand 1 | Cx3cl1 | 1.25 | 0.31 | 1.07 | 0.85 | -1.45 | 0.69 | -1.81 | 0.48 |
| H10 | CD38 antigen | Cd38 | 1.29 | 0.17 | 1.14 | 0.33 | -1.08 | 0.70 | -1.41 | 0.30 |
| H11 | Chemokine (C-X3-C motif) receptor 1 | Cx3cr1 | 1.34 | 0.15 | 1.02 | 0.97 | -1.20 | 0.84 | 1.57 | 0.21 |
| H12 | Positive PCR Control | PPC | -1.05 | 0.68 | -1.16 | 0.78 | 2.05 | 0.34 | 3.69 | 0.29 |

**Supplementary Table 1.** PCR array data of brain tissue (frontal cortex and hippocampus combined) from surgical control, tumor-bearing and tumor-resected mice harvested during the light and/or dark phases (**Fig. 1**). Fold regulation of gene expression compared to the respective surgical control (Light/Dark) was calculated by the 2–ΔΔCt equation and analyzed by two-tailed Student’s t-test. Fold-regulation values greater than 2 highlighted in red; fold-regulation values less than -2 highlighted in blue; p values < 0.05 indicated in red (n = 3-4/group).

**SUPPLEMENTARY TABLE 2**

| **Position** | **Gene Description** | **Gene Symbol** | **Up-Down Regulation (compared to the same group during the dark phase)** | | | | | |
| --- | --- | --- | --- | --- | --- | --- | --- | --- |
|  |  |  | **Surgical Control** | | **Tumor** | | **Tumor-resected** | |
|  |  |  | **Fold Regulation** | **p-value** | **Fold Regulation** | **p-value** | **Fold Regulation** | **p-value** |
| A01 | Serum amyloid P-component | Apcs | 1.06 | 0.90 | 1.69 | 0.34 | 11.02 | 0.29 |
| A02 | Complement component 3 | C3 | -2.13 | **0.03** | 3.33 | 0.06 | 2.26 | 0.34 |
| A03 | Complement component 5a receptor 1 | C5ar1 | 1.44 | 0.88 | 1.14 | 0.59 | 6.65 | 0.28 |
| A04 | Caspase 1 | Casp1 | 1.20 | 0.43 | 1.57 | 0.35 | 3.77 | 0.28 |
| A05 | Chemokine (C-C motif) ligand 12 | Ccl12 | 2.24 | 0.40 | 1.35 | 0.42 | 7.41 | 0.29 |
| A06 | Chemokine (C-C motif) ligand 5 | Ccl5 | -2.01 | 0.24 | -1.63 | 0.16 | 5.38 | 0.29 |
| A07 | Chemokine (C-C motif) receptor 4 | Ccr4 | 1.13 | 0.83 | 2.14 | 0.27 | 13.16 | 0.29 |
| A08 | Chemokine (C-C motif) receptor 5 | Ccr5 | -2.08 | 0.99 | 1.24 | 0.49 | 5.27 | 0.25 |
| A09 | Chemokine (C-C motif) receptor 6 | Ccr6 | -1.54 | 0.41 | 1.38 | 0.68 | 3.98 | 0.28 |
| A10 | Chemokine (C-C motif) receptor 8 | Ccr8 | 1.00 | 0.96 | 1.06 | 0.57 | 8.07 | 0.29 |
| A11 | CD14 antigen | Cd14 | -1.30 | 0.33 | 1.30 | 0.43 | 3.19 | 0.28 |
| A12 | CD4 antigen | Cd4 | -4.15 | **0.04** | -1.08 | 0.43 | -1.03 | 0.36 |
| B01 | CD40 antigen | Cd40 | -1.22 | 0.74 | 1.27 | 0.44 | 2.40 | 0.30 |
| B02 | CD40 ligand | Cd40lg | -1.07 | 0.61 | 1.86 | 0.36 | 7.91 | 0.29 |
| B03 | CD80 antigen | Cd80 | -1.32 | 0.52 | -1.12 | 0.82 | 4.98 | 0.28 |
| B04 | CD86 antigen | Cd86 | 1.16 | 0.36 | -1.00 | 0.99 | 4.74 | 0.27 |
| B05 | CD8 antigen, alpha chain | Cd8a | 1.13 | 0.79 | 1.33 | 0.95 | 22.33 | 0.28 |
| B06 | C-reactive protein, pentraxin-related | Crp | 1.03 | 0.85 | 1.22 | 0.79 | 9.75 | 0.29 |
| B07 | Colony stimulating factor 2 (granulocyte-macrophage) | Csf2 | 1.10 | 0.74 | 1.62 | 0.50 | 8.76 | 0.29 |
| B08 | Chemokine (C-X-C motif) ligand 10 | Cxcl10 | -1.44 | 0.32 | 1.71 | 0.32 | 3.32 | 0.29 |
| B09 | Chemokine (C-X-C motif) receptor 3 | Cxcr3 | -1.51 | 0.28 | 2.23 | 0.42 | 7.61 | 0.29 |
| B10 | DEAD (Asp-Glu-Ala-Asp) box polypeptide 58 | Ddx58 | -1.29 | 0.13 | -1.68 | 0.47 | 2.67 | 0.26 |
| B11 | Fas ligand (TNF superfamily, member 6) | Fasl | 2.34 | 0.09 | 2.18 | 0.06 | 8.30 | 0.28 |
| B12 | Forkhead box P3 | Foxp3 | -1.14 | 0.61 | -1.61 | **0.01** | 5.56 | 0.28 |
| C01 | GATA binding protein 3 | Gata3 | 2.76 | 0.36 | -1.54 | 0.46 | 26.45 | 0.28 |
| C02 | Histocompatibility 2, Q region locus 10 | H2-Q10 | -1.21 | 0.78 | 1.52 | **0.04** | 11.34 | 0.28 |
| C03 | Histocompatibility 2, T region locus 23 | H2-T23 | -1.20 | 0.29 | 1.39 | 0.37 | 2.49 | 0.26 |
| C04 | Intercellular adhesion molecule 1 | Icam1 | 1.17 | 0.33 | 1.03 | 0.44 | 1.11 | 0.37 |
| C05 | Interferon alpha 2 | Ifna2 | -1.14 | 0.83 | 1.15 | 0.71 | 7.56 | 0.29 |
| C06 | Interferon (alpha and beta) receptor 1 | Ifnar1 | -1.09 | 0.64 | -1.65 | 0.30 | 1.58 | 0.23 |
| C07 | Interferon beta 1, fibroblast | Ifnb1 | 1.18 | 0.74 | 3.24 | 0.07 | -2.24 | 0.29 |
| C08 | Interferon gamma | Ifng | 1.32 | 0.62 | -1.56 | 0.85 | 13.75 | 0.29 |
| C09 | Interferon gamma receptor 1 | Ifngr1 | -1.22 | 0.58 | -1.07 | 0.80 | 1.67 | 0.29 |
| C10 | Interleukin 10 | Il10 | -1.01 | 0.99 | 1.93 | 0.34 | 10.40 | 0.29 |
| C11 | Interleukin 13 | Il13 | -1.18 | 0.81 | -1.09 | 0.92 | 16.90 | 0.29 |
| C12 | Interleukin 17A | Il17a | 1.06 | 0.90 | 1.95 | 0.31 | 6.45 | 0.29 |
| D01 | Interleukin 18 | Il18 | -1.03 | 0.85 | 1.24 | 0.44 | 1.85 | 0.32 |
| D02 | Interleukin 1 alpha | Il1a | -1.18 | 0.36 | -1.23 | 0.89 | 1.00 | 0.237 |
| D03 | Interleukin 1 beta | Il1b | -1.66 | 0.25 | -1.16 | 0.89 | -1.76 | 0.29 |
| D04 | Interleukin 1 receptor, type I | Il1r1 | 1.02 | 0.86 | -1.07 | 0.64 | -1.03 | 0.43 |
| D05 | Interleukin 2 | Il2 | 1.06 | 0.90 | 1.40 | 0.44 | 12.20 | 0.29 |
| D06 | Interleukin 23, alpha subunit p19 | Il23a | 1.30 | 0.38 | 2.19 | 0.10 | 13.68 | 0.28 |
| D07 | Interleukin 4 | Il4 | -1.44 | 0.85 | 1.33 | 0.40 | 12.57 | 0.28 |
| D08 | Interleukin 5 | Il5 | 1.22 | 0.53 | -1.13 | 0.54 | 8.33 | 0.29 |
| D09 | Interleukin 6 | Il6 | -1.38 | 0.42 | 1.76 | 0.30 | 12.66 | 0.28 |
| D10 | Interleukin-1 receptor-associated kinase 1 | Irak1 | -1.14 | 0.39 | 1.06 | 0.74 | 1.30 | 0.52 |
| D11 | Interferon regulatory factor 3 | Irf3 | 1.18 | 0.46 | 1.53 | 0.28 | 2.12 | 0.26 |
| D12 | Interferon regulatory factor 7 | Irf7 | -1.35 | 0.32 | -1.32 | 0.27 | 2.17 | 0.30 |
| E01 | Integrin alpha M | Itgam | -1.19 | 0.15 | -1.01 | 0.19 | -1.14 | 0.37 |
| E02 | Janus kinase 2 | Jak2 | 1.07 | 0.71 | -1.02 | 0.86 | 1.78 | 0.17 |
| E03 | Lymphocyte antigen 96 | Ly96 | -1.12 | **0.03** | -1.16 | **0.05** | 1.01 | 0.30 |
| E04 | Lysozyme 2 | Lyz2 | -1.33 | 0.32 | 1.43 | 0.20 | 1.41 | 0.33 |
| E05 | Mitogen-activated protein kinase 1 | Mapk1 | 1.04 | 0.73 | 1.07 | 0.71 | 1.17 | 0.14 |
| E06 | Mitogen-activated protein kinase 8 | Mapk8 | 1.06 | 0.69 | 1.09 | 0.51 | 1.70 | 0.23 |
| E07 | Mannose-binding lectin (protein C) 2 | Mbl2 | 1.70 | 0.28 | 1.57 | 0.51 | 12.25 | 0.29 |
| E08 | Myeloperoxidase | Mpo | 1.99 | 0.06 | 1.90 | 0.18 | -3.12 | 0.29 |
| E09 | Myxovirus (influenza virus) resistance 1 | Mx1 | -2.90 | 0.28 | 1.44 | 0.86 | 8.31 | 0.29 |
| E10 | Myeloid differentiation primary response gene 88 | Myd88 | -1.43 | **0.03** | -1.31 | 0.07 | 1.58 | 0.36 |
| E11 | Nuclear factor of kappa light polypeptide gene enhancer in B-cells 1, p105 | Nfkb1 | -1.01 | 0.89 | 1.05 | 0.62 | 2.04 | 0.28 |
| E12 | Nuclear factor of kappa light polypeptide gene enhancer in B-cells inhibitor, alpha | Nfkbia | 1.34 | **0.01** | 1.75 | 0.19 | 2.34 | 0.26 |
| F01 | NLR Family, pyrin domain containing 3 | Nlrp3 | -1.32 | 0.25 | -1.47 | **0.04** | 3.48 | 0.29 |
| F02 | Nucleotide-binding oligomerization domain containing 1 | Nod1 | 4.98 | 0.36 | -1.27 | 0.34 | 1.39 | 0.38 |
| F03 | Nucleotide-binding oligomerization domain containing 2 | Nod2 | -1.77 | 0.08 | -1.26 | 0.49 | 8.04 | 0.29 |
| F04 | Recombination activating gene 1 | Rag1 | -1.03 | 0.77 | 2.48 | 0.07 | 7.14 | 0.29 |
| F05 | RAR-related orphan receptor gamma | Rorc | -1.16 | 0.43 | 1.25 | 0.21 | 3.93 | 0.28 |
| F06 | Solute carrier family 11 (proton-coupled divalent metal ion transporters), member 1 | Slc11a1 | -1.12 | 0.41 | 1.05 | 0.82 | 3.65 | 0.28 |
| F07 | Signal transducer and activator of transcription 1 | Stat1 | -1.00 | 0.83 | -1.37 | 0.19 | 1.80 | 0.28 |
| F08 | Signal transducer and activator of transcription 3 | Stat3 | -1.07 | 0.18 | -1.09 | 0.33 | 1.52 | 0.21 |
| F09 | Signal transducer and activator of transcription 4 | Stat4 | -1.35 | 0.31 | -1.41 | **0.04** | 2.78 | 0.30 |
| F10 | Signal transducer and activator of transcription 6 | Stat6 | -1.17 | 0.45 | -1.10 | 0.48 | 2.55 | 0.29 |
| F11 | T-box 21 | Tbx21 | -1.46 | 0.43 | 1.29 | 0.43 | 5.64 | 0.28 |
| F12 | Toll-like receptor adaptor molecule 1 | Ticam1 | 1.05 | 0.85 | -1.82 | 0.09 | 2.58 | 0.23 |
| G01 | Toll-like receptor 1 | Tlr1 | -1.03 | 0.95 | 1.22 | 0.30 | 1.98 | 0.32 |
| G02 | Toll-like receptor 2 | Tlr2 | -1.05 | 0.80 | -1.80 | 0.33 | 4.02 | 0.28 |
| G03 | Toll-like receptor 3 | Tlr3 | -1.13 | 0.57 | -1.05 | 0.82 | -1.35 | 0.32 |
| G04 | Toll-like receptor 4 | Tlr4 | -1.04 | 0.88 | 1.21 | 0.46 | -1.15 | 0.29 |
| G05 | Toll-like receptor 5 | Tlr5 | -1.90 | 0.11 | 1.17 | 0.59 | 5.29 | 0.29 |
| G06 | Toll-like receptor 6 | Tlr6 | 1.22 | 0.75 | -1.47 | 0.22 | 3.23 | 0.28 |
| G07 | Toll-like receptor 7 | Tlr7 | -1.06 | 0.62 | 1.47 | 0.38 | -1.05 | 0.35 |
| G08 | Toll-like receptor 8 | Tlr8 | 1.47 | 0.65 | 1.38 | 0.89 | 6.74 | 0.29 |
| G09 | Toll-like receptor 9 | Tlr9 | -1.47 | **0.03** | -1.85 | **0.03** | 1.15 | 0.40 |
| G10 | Tumor necrosis factor | Tnf | -2.23 | 0.25 | -1.06 | 0.82 | 12.04 | 0.28 |
| G11 | Tnf receptor-associated factor 6 | Traf6 | 1.03 | 0.84 | -1.33 | 0.52 | 1.19 | 0.37 |
| G12 | Tyrosine kinase 2 | Tyk2 | -1.27 | 0.18 | -1.41 | 0.31 | 1.23 | 0.45 |
| H01 | Actin, beta | Actb | -1.07 | 0.26 | -1.06 | 0.72 | -1.45 | 0.14 |
| H02 | Beta-2 microglobulin | B2m | 1.05 | 0.66 | 1.63 | 0.30 | 1.66 | 0.33 |
| H03 | Glyceraldehyde-3-phosphate dehydrogenase | Gapdh | -1.05 | 0.22 | -1.03 | 0.65 | 1.30 | 0.24 |
| H04 | Glucuronidase, beta | Gusb | -1.04 | 0.82 | 1.19 | 0.43 | 1.95 | 0.26 |
| H05 | Heat shock protein 90 alpha (cytosolic), class B member 1 | Hsp90ab1 | 1.12 | 0.07 | 1.09 | 0.43 | 1.11 | 0.25 |
| H06 | Mouse Genomic DNA Contamination | MGDC | 1.06 | 0.90 | 1.66 | 0.35 | 8.93 | 0.29 |
| H07 | Reverse Transcription Control | RTC | 1.19 | 0.65 | 1.99 | 0.30 | 3.23 | 0.29 |
| H08 | CD68 antigen | Cd68 | 1.05 | 0.65 | 1.30 | 0.38 | 1.61 | 0.34 |
| H09 | Chemokine (C-X3-C motif) ligand 1 | Cx3cl1 | -1.03 | 0.80 | -1.86 | 0.25 | -1.99 | 0.32 |
| H10 | CD38 antigen | Cd38 | 1.43 | **0.02** | 1.16 | 0.51 | -1.14 | 0.42 |
| H11 | Chemokine (C-X3-C motif) receptor 1 | Cx3cr1 | -1.15 | 0.39 | -1.84 | 0.09 | 1.34 | 0.32 |
| H12 | Positive PCR Control | PPC | 1.11 | 0.80 | 2.40 | 0.28 | 4.73 | 0.29 |

**Supplementary Table 2.** PCR array data of brain tissue (frontal cortex and hippocampus combined) from surgical control, tumor-bearing and tumor-resected mice harvested during the light and/or dark phases (**Fig. 1**). Fold regulation of gene expression compared to the respective group during the dark phase was calculated by the 2–ΔΔCt equation and analyzed by two-tailed Student’s t-test. Fold-regulation values greater than 2 highlighted in red; fold-regulation values less than -2 highlighted in blue; p values < 0.05 indicated in red (n = 3-4/group).

**SUPPLEMENTARY FIGURES**


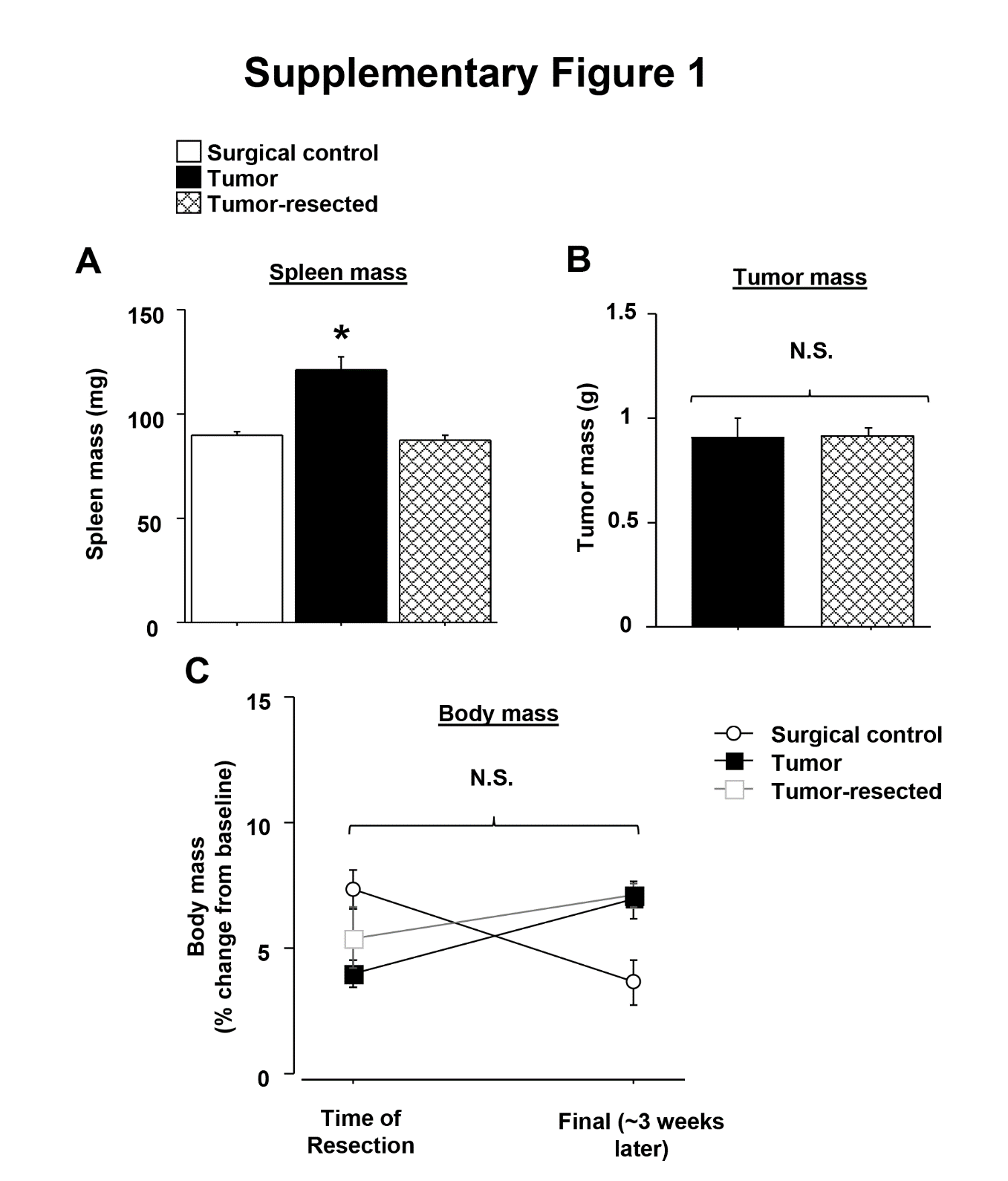


**Supplementary Figure 1. Physiological comparisons of tumor conditions.** A) Mammary tumors significantly increased spleen mass, which was rescued by tumor resection. B) Final tumor mass of tumor-bearing mice was not significantly different from tumor-resected mice at time of resection. C) Body mass did not vary significantly over time between tumor conditions. *: *p* < 0.05 between tumor conditions; ns: not significant by two-way ANOVA.


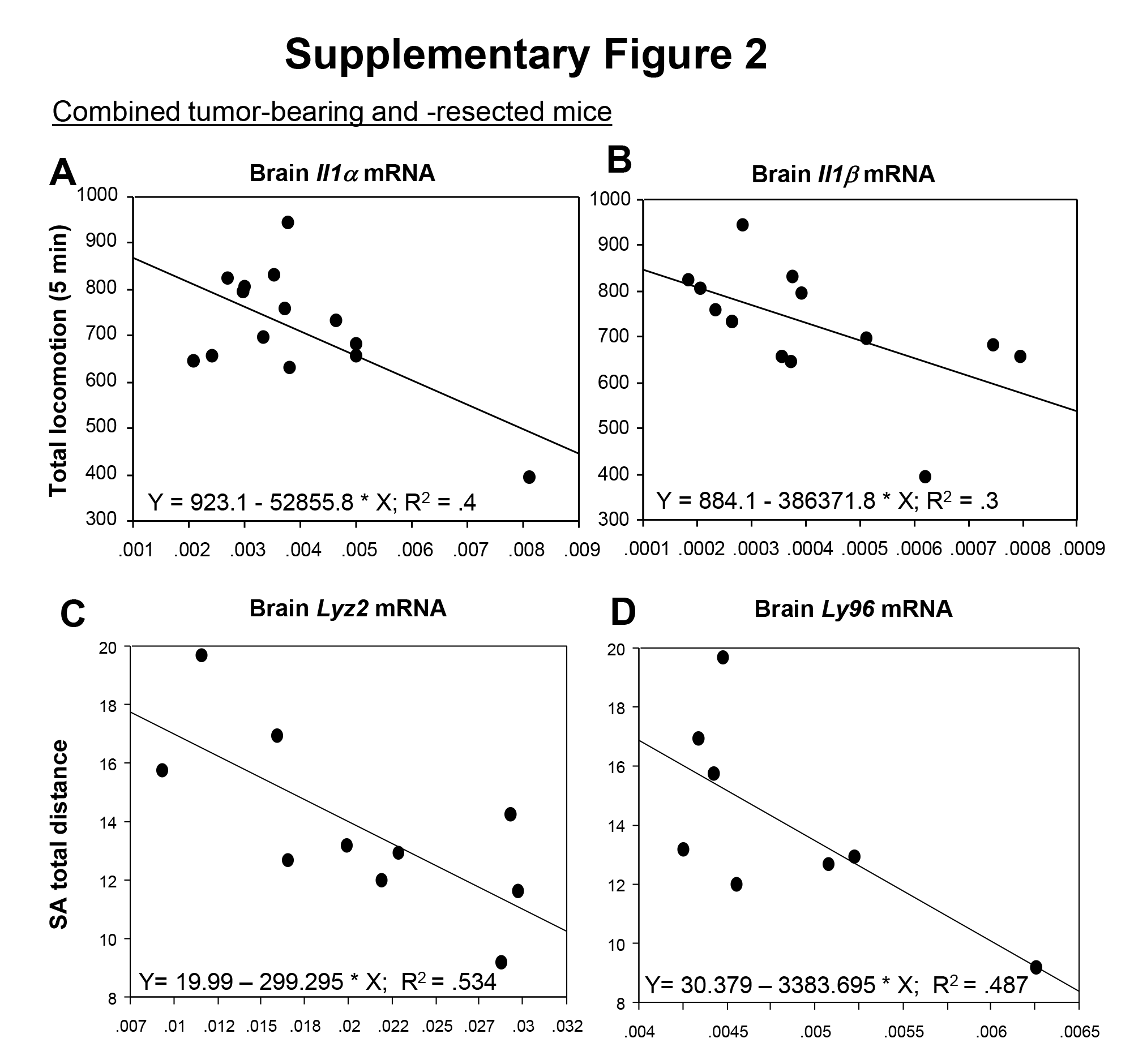


**Supplementary Figure 2. Correlation between open field behavior and neuroinflammation.** Linear regressions between total locomotion and neuroinflammatory mediators mRNA expression in tumor-bearing and -resected mice during the dark phase. Correlation between total locomotion and brain *Il1α A)* and *Il1β* B) mRNA expression in the brain during the dark phase. Correlation between total distance traveled on the spontaneous alternation test and brain *Lyz2* C) and *Ly96* D)mRNA expression in the brain during the dark phase.


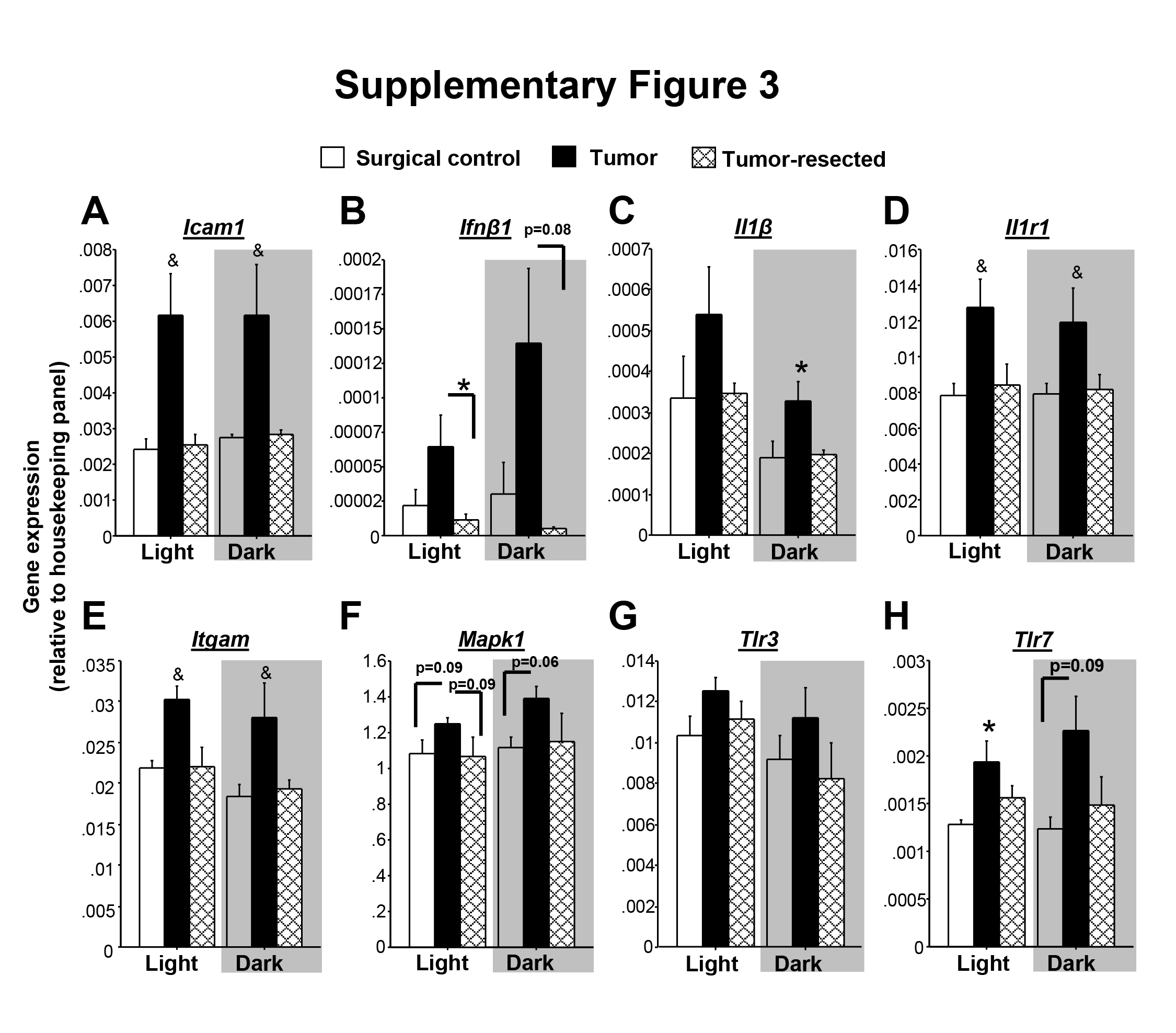


**Supplementary Figure 3. Effects of tumors on neuroimmune gene expression observed during both the light and dark phases.** Mammary tumors significantly increased several immune/inflammatory genes regardless of time of day. Mean ± SEM of gene expression in pooled RNA from hippocampal and frontal cortex tissue, evaluated using the RT^2^ Profiler PCR Array (Biosiences, Qiagen). A) *Icam1*, B) *Ifnβ1*, C) *Il1β,*  D) *Il1r1,* E) *Itgam*, F) *Mapk1,* G)  *Tlr3 and* H) *Tlr7*. *p<0.05 relative to tumor-free mice at the same time-of-day; ^#^p<0.05 relative to tumor-bearing mice at the same time-of-day; ^&^p<0.05 relative to PBS-treated mice by two-way ANOVA.


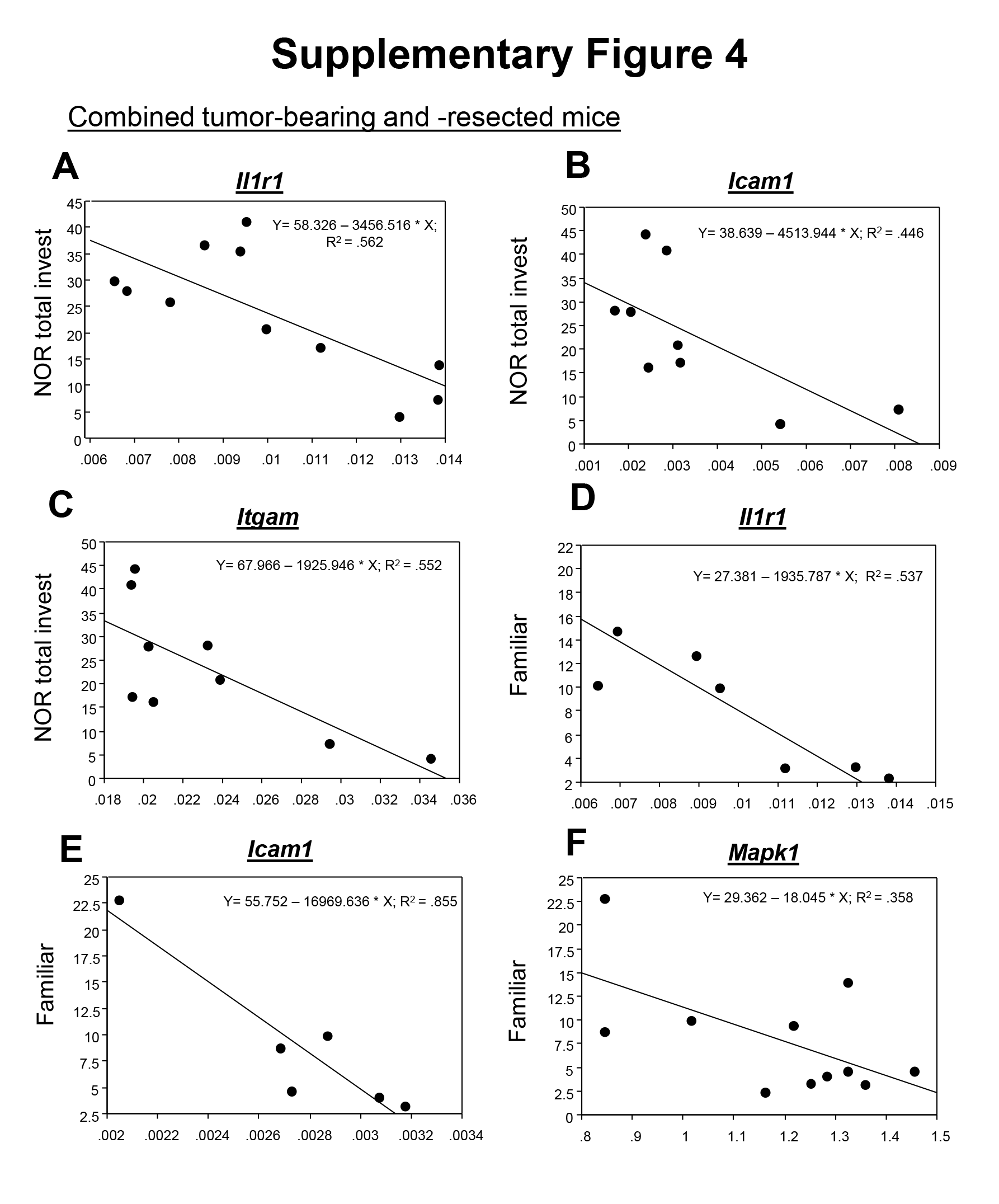


**Supplementary Figure 4. Correlation between novel object recognition behavior and neuroinflammation.** Linear regressions between novel object recognition (NOR) outcomes and brain *Il1r1, Icam1, Itgam or Mapk1* mRNA expression in tumor bearing mice during the dark phase. Correlation between NOR total investigation and brain *Ilr1* A), *Icam1* B) and *Itgam* C) mRNA expression in the brain during the dark phase. Correlation between time spent with the familiar object and brain *Il1r1* D), *Icam1* E) and *Mapk1* F) mRNA expression in the brain during the dark phase.
